# Evaluation of Ruthenium-Based Assemblies as Carriers of Photosensitizers to Treat Rheumatoid Arthritis by Photodynamic Therapy

**DOI:** 10.3390/pharmaceutics13122104

**Published:** 2021-12-07

**Authors:** Manuel Gallardo-Villagrán, Lucie Paulus, Jean-Louis Charissoux, Sylvain Sutour, Pascale Vergne-Salle, David Yannick Leger, Bertrand Liagre, Bruno Therrien

**Affiliations:** 1 Institut de Chimie, Université de Neuchâtel, Avenue de Bellevaux 51, CH-2000 Neuchâtel, Switzerland; manuel.gallardo@unine.ch; 2 Laboratoire PEIRENE EA 7500, Faculté de Pharmacie, Université de Limoges, 87025 Limoges, France; lucie.paulus@etu.unilim.fr (L.P.); david.leger@unilim.fr (D.Y.L.); 3 Service d’Orthopédie-Traumatologie, CHRU Dupuytren, 2 Avenue Martin Luther King, CEDEX, 87042 Limoges, France; jlouis.charissoux@chu-limoges.fr; 4 Neuchâtel Platform for Analytical Chemistry (NPAC), University of Neuchâtel, Avenue de Bellevaux 51, CH-2000 Neuchâtel, Switzerland; sylvain.sutour@unine.ch; 5 Service de Rhumatologie, CHRU Dupuytren 2, 16 rue Bernard Descottes, CEDEX, 87042 Limoges, France; pascale.vergne-salle@chu-limoges.fr

**Keywords:** rheumatoid arthritis, photodynamic therapy, drug delivery, host–guest system, COX-2, photosensitizer, arene ruthenium complexes

## Abstract

For the first time, ruthenium-based assemblies have been used as carriers for photosensitizers in the treatment of rheumatoid arthritis by photodynamic therapy (PDT). These metallacages are totally soluble in physiological media and can transport photosensitizers (PS) in their cavity. After an incubation period, the PS is released in the cytoplasm and irradiation can take place. This strategy allows photosensitizers with low or null solubility in biological media to be evaluated as PDT agents in rheumatoid arthritis. The systems in which 21*H*,23*H*-porphine and 29*H*,31*H*-phthalocyanine are encapsulated show excellent photocytotoxicity and no toxicity in the dark. On the other hand, systems in which metalated derivatives such as Mg(II)-porphine and Zn(II)-phthalocyanine are used show good photocytotoxicity, but to a lesser extent than the previous two. Furthermore, the presence of Zn(II)-phthalocyanine significantly increases the toxicity of the system. Overall, fifteen different host–guest systems have been evaluated, and based on the results obtained, they show high potential for treating rheumatoid arthritis by PDT.

## 1. Introduction

Rheumatoid arthritis (RA) is a chronic inflammatory autoimmune joint disease, leading to cartilage and bone damage, and finally disability. Occasionally, RA is complicated with extra-articular manifestations, particularly pulmonary involvement, and is associated with cardiovascular comorbidities [1]. The prevalence is 0.3% to 1%, and is 2–3 times higher in women than in men [2].

In recent years, it has become evident that RA arises based on both genetic and epigenetic components, but also has an environmental component, such as cigarette smoke, dust exposure, and particularly the effect of the microbiome [3]. Abnormalities in the cellular and humoral immune response lead to the occurrence of autoantibodies, detected many months or years before the clinical disease is apparent. These autoantibodies are rheumatoid factors (directed against the Fc fragment of immunoglobulins) and anti-citrullinated protein antibodies (ACPA) [4,5].

In RA, the synovial lining, which normally is comprised of 1–3 cell layers, becomes remarkably thickened. This is due to an invasion of macrophage-like cells and the proliferation of resident synovial fibroblasts. The degree of synovial hyperplasia correlates with the severity of cartilage erosion, resulting in inflammatory pannus formation that attaches to, and invades, joint cartilage, while osteoclast activation leads to parallel bone destruction [6]. The interaction between synovial resident cells and cells of the innate and adaptative immune system leads to the production of many pro-inflammatory cytokines (TNF-α, interleukine-1 (IL-1), IL-6), proteolytic enzymes, and inflammatory molecules [1].

Treatment algorithms involve measuring disease activity with composite indices and applying a treatment-to-target strategy, with disease modifying antirheumatic drugs (DMARDs) to maintain stringent remission or at least low disease activity and reduce articular destruction and disability [7]. There are two major classes of DMARDs, namely synthetic (sDMARDs) and biological (bDMARDs) [8].

Despite all of the advances made over the last two decades, and given that remission or at least low disease activity are the current therapeutic goals for RA patients, a significant proportion of patients still do not reach this target. There is a need for new treatments or local treatments to control some resistant synovitis.

In recent years, promising results have been achieved using non-invasive treatments such as anti-tumor necrosis factor drugs [9], Janus kinase inhibitors [10], and photodynamic therapy (PDT) [11]. The latter involves a photoactive compound, termed the photosensitizer (PS), which is excited by suitable light radiation. Subsequently, the excitation energy gives rise to radical oxygen species (ROS) from oxygen present in the medium [12,13]. ROS show a high cytotoxicity, but also a short lifetime and reduced radius of action [14], so it is possible to treat the inflamed zone without affecting the surrounding tissue, avoiding damage to healthy structures. Accordingly, PDT could be an effective solution for cases of RA with refractory synovitis and failure of local steroid injection.

Since the late 1990s, PDT began to demonstrate its potential as a less invasive treatment for RA. Trauner and colleagues [15] reported the in vivo efficacy of this technique in rabbits with an antigen-induced arthritis model, using benzoporphyrin derivative monoacid ring A (one of the first-generation PSs) and intra-articular irradiation. Later, with the new generation PSs, in vivo results remained promising, such as the use of ATX-S10.Na (II) in collagen antibody-induced arthritis in mice [16]. Recently, it has been reported that the combined use of photothermal therapy and PDT using Cu_7.2_S_4_ nanoparticles under NIR laser in mice, improves anti-inflammatory effects and reduces cartilage and bone damage [17].

**Figure 1:**
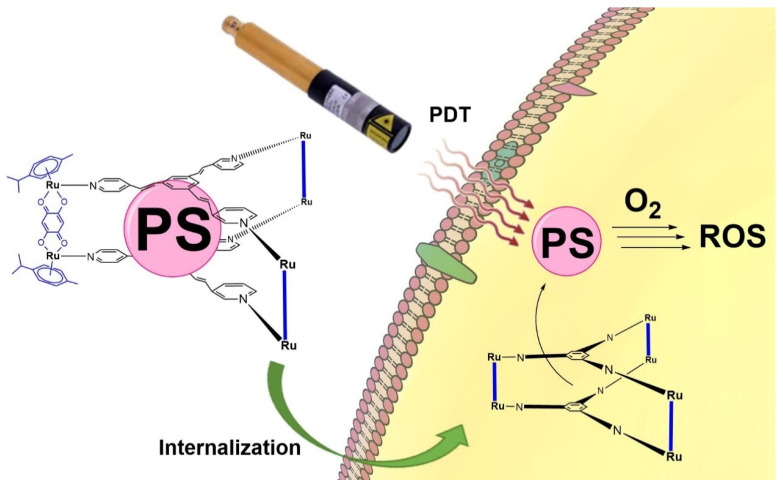
Typical ruthenium-based assemblies used as PS carrier for cellular internalization and subsequent activation of PS by irradiation, giving rise to ROS.

The simplicity of the technique makes PDT an ideal treatment to alleviate the pain or disability caused by RA. Unfortunately, even considering the enormous potential of PDT, conventional PSs often have some drawbacks mainly related to their chemical and structural features, as well as undesirable side effects in some cases, such as light hypersensitivity [18]. Most recent studies have focused on solving the poor solubility of PSs in biological media using soluble carriers [19] such as nanoparticles or by adding a soluble functional group in the PS structure [20]. We believe that it may be possible to solve the poor water solubility of PSs using another approach: ruthenium-based carriers (Figure 1). These organometallic complexes are soluble in biological media and have an inner cavity in which a PS can be lodged. Such metallacages have already been tested in vitro on cancer cells, demonstrating their potential in cells [21,22].

**Figure 2:**
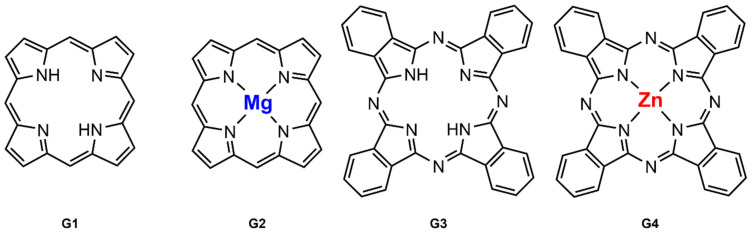
Photosensitizers used in this work. From left to right, 21H,23H-porphine (**G1**), Mg(II)-porphine (**G2**), 29H,31H-phthalocyanine (**G3**) and Zn(II)-phthalocyanine (**G4**).

For the first time, we showed that such carriers can be used as PDT agents in fibroblast-like synoviocyte cells (FLS) from RA patients. We also demonstrated that commercially available PSs (Figure 2), namely 21*H*,23*H*-porphine (**G1**), Mg(II)-porphine (**G2**), 29*H*,31*H*-phthalocyanine (**G3**), and zinc(II)-phthalocyanine (**G4**), can be encapsulated in the cavity of the metallacages and, after being released, become effective PSs against RA. We have hosted these basic PSs in different ruthenium-based assemblies, showing the importance of the carrier in delivering the PS. The in vitro evaluation of these PS-metallacages in human RA FLS cells is promising. The anti-proliferative assays are excellent, providing new avenues for the treatment of RA by PDT.

## 2. Materials and Methods

### 2.1. Synthesis of Compounds

**Figure 3:**
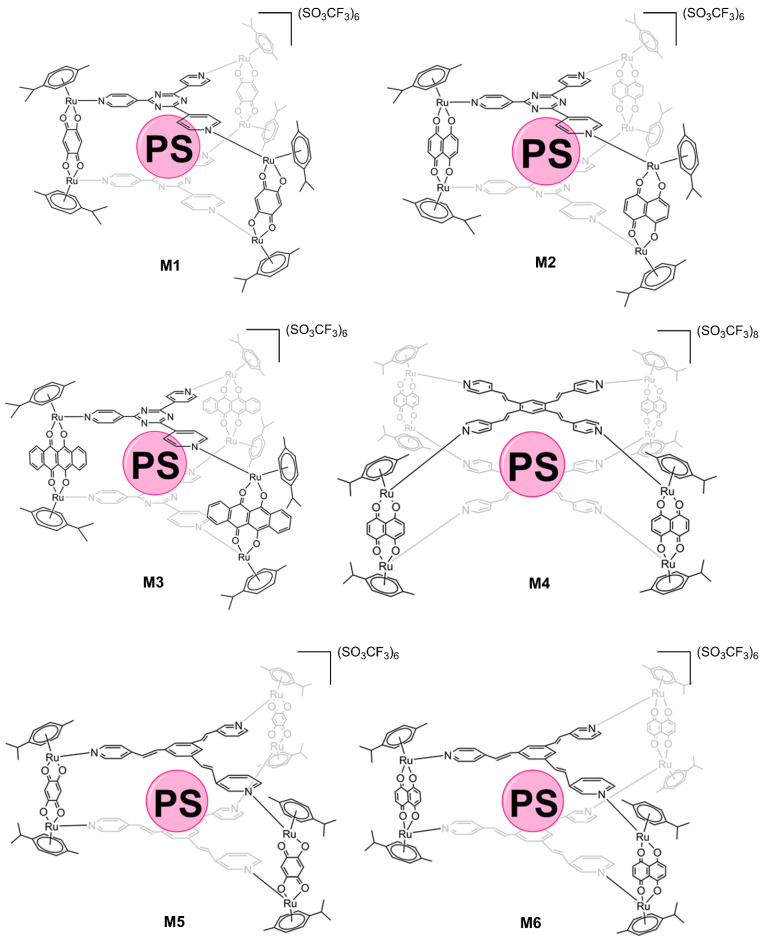
Structures of ruthenium(II) metallacages used in this work. The photosensitizer is represented by a sphere (PS), 21*H*,23*H*-porphine (**G1**) was inserted in **M1**–**M6**, Mg(II)-porphine (**G2**) in **M1**, **M4,** and **M6**, 29*H*,31*H*-phthalocyanine (**G3**) and Zn(II)-phthalocyanine (**G4**) in **M4**–**M6**.

Despite the fact that the hosts **M1**-**M6** (Figure 3) and the guests **G1**-**G4** are known, several host–guest systems are new, except for **G1**⊂**M2**, **G1**⊂**M3**, **G2**⊂**M1**, **G2**⊂**M4**, and **G2**⊂**M6**. The complexes [Ru_2_(*p*-cymene)_2_(2,5-dioxydo-1,4-benzoquinonato)Cl_2_], [Ru_2_(*p*-cymene)_2_(5,8-dioxydo-1,4-naphthoquinonato)Cl_2_], [Ru_2_(*p*-cymene)_2_(6,11-dioxydo-5,12-naphthacenedionato)Cl_2_] [23], and the ligands 2,4,6-tris(pyridin-4-yl)-1,3,5-triazine [24], 1,3,5-tris{2-(pyridin-4-yl)vinyl} benzene [25], and 1,2,4,5-tetrakis{2-(pyridine-4-yl)vinyl} benzene [26] were prepared following reported methods. The metallacages **M1** and **M4** with **G1** inside [21], **M4** with **G3** and **G4**, **M5** and **M6** with **G1**, **G3**, and **G4** [27] were synthesized according to the literature. The photosensitizers **G1** and **G2** were synthesized as reported in the literature [28], while **G3** and **G4** were bought from Sigma-Aldrich. Dichloromethane, diethyl ether, methanol, *d*_3_-acetonitrile, and *d*_6_-DMSO were purchased from Sigma-Aldrich and used as received. NMR spectra were measured on a Bruker Avance Neo Ascend 600 MHz spectrometer. The acquired spectra were processed using the Mnova NMR software package (v.14.2.0, MestReLab Research, Santiago de Compostela, Spain). The ^1^H and ^13^C resonances of the deuterated solvents were used as internal references. The following abbreviations are used for describing the signals in the NMR spectra: s (singlet), d (doublet), m (multiplet), br (broad), q (quaternary). All described in vitro experiments were carried out under aseptic conditions. 3-(4,5-Dimethylthiazol-2-yl)-2,5-diphenyltetrazolium bromide (MTT) and L-glutamine were purchased from Sigma-Aldrich (St. Louis, MO, USA). Dimethyl sulfoxide (DMSO) was bought from Acros Organics (Geel, Belgium). All solvents, reagents, and products described above were used without prior treatments or purifications. IR spectra of the compounds were performed on a Frontier Perkin Elmer spectrometer (600–4000 cm^−1^), Thermo Fisher Scientific, Waltham, MA, USA. Fluorescence spectra were performed on a FLS980 spectrometer from Edinburgh instruments (550–800 nm) using 5,10,15,20-tetraphenylporphin (TPP) as an internal reference in toluene and the compounds were dissolved in DMSO (10 nM concentration). UV-vis spectra were acquired on a SI Analytics model UvLine 9400 (Xenon lamp) spectrophotometer, using 1.5 mL polystyrene cuvettes (wavelength range 280–800 nm) and diluting the compounds in DMSO (10 µM and 10 nM).

**Synthesis of G1**⊂**M2**. In a 250 mL round bottom flask, 50.0 mg (0.069 mmol) of [Ru_2_(*p*-cymene)_2_(5,8-dioxydo-1,4-naphthoquinonato)Cl_2_] and 35.5 mg (0.138 mmol) of AgCF_3_SO_3_ were dissolved in 20 mL of MeOH and stirred for 2 h at room temperature. Next, silver chloride was filtered off. To the remaining solution, 14.6 mg (0.046 mmol) of 2,4,6-tri(pyridin-4-yl)-1,3,5-triazine and 7.1 mg (0.023 mmol) of **G1** were added, and the solution was refluxed and stirred for 18 h. The solvent was then removed at reduced pressure, and the resulting oily, dark green solid was dissolved in 20 mL of CH_2_Cl_2_. The solution was concentrated to approximately 3 mL, and 5 mL of Et_2_O was added dropwise. The resulting precipitate was filtered and dried under vacuum. Yield 52% (45 mg). ^1^H NMR (CD_3_CN, 25 °C, 600 MHz): δ 9.17 (d, ^3^*J_HH_* = 6.0 Hz, 12H, C*H*_naphce_), 8.44 (d, ^3^*J_HH_* = 4.1 Hz, 12H, C*H*_naphce_), 8.24 (d, ^3^*J_HH_* = 6.3 Hz, 12H, C*H*_py_), 8.00 (s, 4H, C*H*_porphine_), 6.89 (s, 8H, C*H*_porphine_), 5.91 (d, ^3^*J_HH_* = 6.4 Hz, 12H, C*H*_py_), 5.87 (d, ^3^*J_HH_* = 5.9 Hz, 12H, C*H*_cym_), 5.57 (d, ^3^*J_HH_* = 5.8 Hz, 12H, C*H*_cym_), 2.95 (m, 6H, C*H*_iPr_), 1.97 (overlapped singlet, 18H, C*H*_3_), 1.32 (d, ^3^*J_HH_* = 7.0 Hz, 36H, CH_3 iPr_). ^13^C NMR (CD_3_CN, 25 °C, 150 MHz): δ 170.4 (*C*-O), 164.3 (*C*_q_), 152.1 (*C*H_py_), 140.7 (*C*_q_), 134.6 (*C*H_naphce_), 130.1 (*C*H_porph_), 128.4 (*C*H_naphce_), 122.8 (*C*_q_), 122.3 (*C*H_py_), 120.7 (*C*_q_), 107.9 (*C*_q_), 104.4 (*C*_cym_), 103.3 (*C*H_porph_), 100.3 (*C*_cym_), 84.9 (*C*H_cym_), 83.0 (*C*H_cym_), 31.0 (*C*H_iPr_), 22.0 (*C*H_3 iPr_), 17.3 (*C*H_3_). ESI-MS, m/z, 1120 [**M2**+**G1**-3OTf]^3+^. UV/vis (DMSO), λ, nm (ε, M^−1^·cm^−1^): 454 (132400), 488 (117700), 567 (54300), 648 (62900). FT-IR (ATR, solid, cm^−1^): ν; br s (3700–3100), s (2995), s (1524), s (1516). Spectra in Supplementary Materials (Figures S1–S9).

**Synthesis of G1**⊂**M3**. In a 250 mL round bottom flask, 50.0 mg (0.060 mmol) of [Ru_2_(*p*-cymene)_2_(6,11-dioxydo-5,12-naphthacenedionato)Cl_2_] and 31.0 mg (0.120 mmol) of AgCF_3_SO_3_ were dissolved in 20 mL of MeOH and stirred for 2 h at room temperature. Next, silver chloride was filtered off. To the remaining solution, 12.5 mg (0.040 mmol) of 2,4,6-tri(pyridine-4-yl)-1,3,5-triazine and 6.2 mg (0.020 mmol) of **G1** were added, and the solution was refluxed and stirred for 18 h. The solvent was then removed at reduced pressure, and the resulting oily, dark green solid was dissolved in 20 mL of CH_2_Cl_2_. The solution was concentrated to approximately 3 mL, and 5 mL of Et_2_O was added dropwise. The resulting precipitate was filtered and dried under vacuum. Yield 76% (62 mg). ^1^H NMR (CD_3_CN, 25 °C, 600 MHz): δ 8.68 (s, 4H, C*H*_porphine_), 8.21 (d, ^3^*J_HH_* = 6.3 Hz, 12H, C*H*_py_), 7.92 (s, 12H, C*H*_naph_), 7.71 (s, 8H, C*H*_porphine_), 6.12 (d, ^3^*J_HH_* = 5.7 Hz, 12H, C*H*_py_), 5.68 (d, ^3^*J_HH_* = 5.8 Hz, 12H, C*H*_cym_), 5.43 (d, ^3^*J_HH_* = 5.9 Hz, 12H, C*H*_cym_), 2.84 (m, ^3^*J_HH_* = 6.8 Hz, 6H, C*H*_iPr_), 1.99 (s, 18H, C*H*_3_), 1.33 (d, ^3^*J_HH_* = 6.9 Hz, 36H, CH_3 iPr_). ^13^C NMR (CD_3_CN, 25 °C, 150 MHz): δ 171.9 (*C*-O), 164.5 (*C*_q_), 152.3 (*C*H_py_), 140.9 (*C*_q_), 138.9 (*C*H_naph_), 130.9 (*C*H_porph_), 124.9 (*C*_q_), 122.8 (*C*H_py_), 122.5 (*C*H_py_), 120.7 (*C*_q_), 112.5 (*C*_q_), 104.4 (*C*_cym_), 103.9 (*C*H_porph_), 100.3 (*C*_cym_), 85.0 (*C*H_cym_), 83.7 (*C*H_cym_), 31.0 (*C*H_iPr_), 21.9 (*C*H_3 iPr_), 16.8 (*C*H_3_). UV/vis (DMSO), λ, nm (ε, M^−1^·cm^−1^): 489 (53700), 573 (40200), 623 (62900). FT-IR (ATR, solid, cm^−1^): ν; br s (3700–3100), br s (3092), s (2992), s (2915), s (1531), s (1502). Spectra in Supplementary Materials (Figures S10–S17).

**Synthesis of G2**⊂**M1.** In a 250 mL round bottom flask, 50 mg (0.074 mmol) of [Ru_2_(*p*-cymene)_2_(2,5-dioxydo-1,4-benzoquinonato)Cl_2_] and 37.8 (0.148 mmol) mg of AgCF_3_SO_3_ were dissolved in 20 mL of MeOH and stirred for 2 h at room temperature. Next, silver chloride was filtered off. To the remaining solution, 15.4 mg (0.049 mmol) of 2,4,6-tri(pyridin-4-yl)-1,3,5-triazine and 8.1 mg (0.025 mmol) of **G2** were added, and the solution was refluxed and stirred for 18 h. The solvent was then removed at reduced pressure, and the resulting oily, dark green solid was dissolved in 20 mL of CH_2_Cl_2_. The solution was concentrated to approximately 3 mL, and 5 mL of Et_2_O was added dropwise. The resulting precipitate was filtered and dried under vacuum. Yield 64% (59 mg). ^1^H NMR (DMSO-d6, 25 °C, 600 MHz): δ 10.42 (s, 4H, C*H*_Mg-porphine_), 9.61 (s, 8H, C*H*_Mg-porphine_), 8.57 (m, 24H, C*H*_py_), 5.62 (d, ^3^*J_HH_* = 6.1 Hz, 12H, C*H*_cym_), 5.98 (d, ^3^*J_HH_* = 6.1 Hz, 12H, C*H*_cym_), 5.91 (s, 6H, C*H*_bz_), 2.82 (m, 6H, C*H*_iPr_), 2.08 (s, 18H, C*H*_3_), 1.28 (d, ^3^*J_HH_* = 6.9 Hz, 36H, CH_3 iPr_). ^13^C NMR (DMSO-d6, 25 °C, 150 MHz): δ 184.1 (*C*-O), 169.4 (*C*_q_), 154.5 (*C*H_py_), 149.3 (*C*_q_), 144.6 (*C*_q_), 132.7 (*C*H_Mg-porphine_), 129.2 (*C*_q_), 126.5 (*C*_q_), 124.8 (*C*H_py_), 122.2 (*C*_q_), 120.0 (*C*_q_), 105.9 (*C*H_Mg-porphine_), 103.8 (*C*_cym_), 101.8 (*C*H_bz_), 99.7 (*C*_cym_), 84.1 (*C*H_cym_), 81.9 (*C*H_cym_), 31.1 (*C*H_iPr_), 22.4 (*C*H_3 iPr_), 17.9 (*C*H_3_). Elemental analysis: Calcd. For C_140_H_126_F_18_MgN_16_O_30_Ru_6_S_6_ + 6H_2_O: C, 44.42; H, 3.67; N, 5.92. Found: C, 45.23; H, 4.08; N, 6.09. ESI-MS, m/z, 770 [**M1**+**G2**-4OTf]^4+^. UV/vis (DMSO), λ, nm (ε, M^−1^·cm^−1^): 500 (140700), 535 (130400). FT-IR (ATR, solid, cm^−1^): ν; s (3093), s (2977), s (2911), s (2804), s (1508). Spectra in Supplementary Materials (Figures S18–S26).

**Synthesis of G2**⊂**M4.** In a 250 mL round bottom flask, 50 mg (0.069 mmol) of [Ru_2_(*p*-cymene)_2_(5,8-dioxydo-1,4-naphthoquinonato)Cl_2_] and 35.5 mg (0.138 mmol) of AgCF_3_SO_3_ were dissolved in 20 mL of MeOH and stirred for 2 h at room temperature. Next, silver chloride was filtered off. To the remaining solution, 16.81 mg (0.034 mmol) of 1,2,4,5-tetrakis{2-(pyridine-4-yl)vinyl} benzene and 5.7 mg (0.017 mmol) of **G2** were added, and the solution was refluxed and stirred for 18 h. The solvent was then removed at reduced pressure, and the resulting oily, dark green solid was dissolved in 20 mL of CH_2_Cl_2_. The solution was concentrated to approximately 3 mL, and 5 mL of Et_2_O was added dropwise. The resulting precipitate was filtered and dried under vacuum. Yield 59% (52 mg). ^1^H NMR (CD_3_CN, 25 °C, 600 MHz): δ 10.02 (s, 4H, C*H*_Mg-porphine_), 9.13 (s, 8H, C*H*_Mg-porphine_), 8.18 (d, ^3^*J_HH_* = 5.1 Hz, 16H, C*H*_py_), 7.49 (d, ^3^*J_HH_* = 15.1 Hz, 8H, C*H*=C), 7.20 (m, 40H, C*H*_naph_, C*H*_py_, C*H*=C), 6.90 (s, 4H, C*H*_ar_), 5.60 (d, ^3^*J_HH_* = 4.46 Hz, 16H, C*H*_cym_), 5.41 (d, ^3^*J_HH_* = 3.1 Hz, 16H, C*H*_cym_), 2.76 (m, ^3^*J_HH_* = 6.9 Hz, 8H, C*H*_iPr_), 2.03 (overlapped singlet, 24H, C*H*_3_), 1.25 (d, ^3^*J_HH_* = 6.9 Hz, 48H, CH_3 iPr_). ^13^C NMR (CD_3_CN, 25 °C, 150 MHz): δ 171.4 (*C*-O), 152.3 (*C*H_py_), 149.9 (*C*_q_), 147.7 (*C*_q_), 138.1 (*C*H_py_), 128.6 (*C*H=C), 127.6 (*C*H=C), 123.6 (*C*H_naph_), 122.8 (*C*_q_), 112.1 (*C*_q_), 104.1 (*C*_cym_), 99.7 (*C*_cym_), 84.7 (*C*H_cym_), 83.7 (*C*H_cym_), 31.0 (*C*H_iPr_), 21.9 (*C*H_3 iPr_), 16.9 (*C*H_3_). ESI-MS, m/z, 880 [**M4**+**G2**-5OTf]^5+^. UV/vis (DMSO), λ, nm (ε, M^−1^·cm^−1^): 536 (137900), 572 (80300), 610 (64600). FT-IR (ATR, solid, cm^−1^): ν; s (3089), s (2947), s (2911), s (2861), s (1619), (1554). Spectra in Supplementary Materials (Figures S27–S35).

**Synthesis of G2**⊂**M6.** In a 250 mL round bottom flask, 50 mg (0.069 mmol) of [Ru_2_(*p*-cymene)_2_(5,8-dioxydo-1,4-naphthoquinonato)Cl_2_] and 35.5 mg (0.138) of AgCF_3_SO_3_ were dissolved in 20 mL of MeOH and stirred for 2 h at room temperature. Next, silver chloride was filtered off. To the remaining solution, 17.8 mg (0.046 mmol) of panel ligand 1,3,5-tris{2-(pyridin-4-yl)vinyl}benzene and 7.6 mg (0.023 mmol) of **G2** were added, and the solution was refluxed and stirred for 18 h. The solvent was then removed at reduced pressure, and the resulting oily, dark green solid was dissolved in 20 mL of CH_2_Cl_2_. The solution was concentrated to approximately 3 mL, and 5 mL of Et_2_O was added dropwise. The resulting precipitate was filtered and dried under vacuum. Yield 61% (56 mg). ^1^H NMR (CD_3_CN, 25 °C, 600 MHz): δ 10.29 (s, 4H, C*H*_Mg-porphine_), 9.41 (s, 8H, C*H*_Mg-porphine_), 8.57 (d, ^3^*J_HH_* = 5.7 Hz, 12H, C*H*_py_), 7.50 (s, 6H, C*H*_ar_), 7.33 (d, ^3^*J_HH_* = 5.7 Hz, 12H, C*H*_py_), 7.26 (s, 12H, C*H*_naph_), 7.22 (overlapped doublet, 6H, C*H*=C), 6.98 (d, ^3^*J_HH_* = 16.1 Hz, 6H, C*H*=C), 5.69 (d, ^3^*J_HH_* = 5.9 Hz, 12H, C*H*_cym_), 5.48 (d, ^3^*J_HH_* = 4.2 Hz, 12H, C*H*_cym_), 2.84 (m, ^3^*J_HH_* = 7.0 Hz, 6H, C*H*_iPr_), 2.10 (s, 18H, C*H*_3_), 1.33 (d, ^3^*J_HH_* = 7.0 Hz, 36H, CH_3 iPr_). ^13^C NMR (CD_3_CN, 25 °C, 150 MHz): δ 171.3 (*C*-O), 152.3 (*C*H_py_), 147.9 (*C*_q_), 138.0 (*C*H_py_), 137.0 (*C*H*_naph_*), 135.1 (*C*H*_naph_*), 132.5 (*C*H_Mg-Porphine_), 127.4 (*C*H=C), 125.2 (*C*H=C), 123.1 (*C*H_ar_), 120.6 (*C*_q_), 112.0 (*C*_q_), 105.9 (*C*H_Mg-porphine_), 104.0 (*C*_cym_), 99.7 (*C*_cym_), 84.6 (*C*H_cym_), 83.5 (*C*H_cym_), 31.0 (*C*H_iPr_), 21.9 (*C*H_3 iPr_), 16.9 (*C*H_3_). Elemental analysis: Calcd. For C_170_H_150_F_18_MgN_10_O_30_Ru_6_S_6_ + 6CH_2_Cl_2_: C, 46.49; H, 3.62; N, 3.06. Found: C, 45.47; H, 3.51; N, 3.88. ESI-MS, m/z, 1177 [**M6**+**G2**-3OTf]^3+^. UV/vis (DMSO), λ, nm (ε, M^−1^·cm^−1^): 446 (150800), 536 (110500), 573 (53500), 610 (47100). FT-IR (ATR, solid, cm-1): ν; s (3101), s (2979), s (2914), s (1604), s (1522). Spectra in Supplementary Materials (Figures S36–S43).

### 2.2. Preparation of Human Synovial Cells

RA synoviocytes were isolated from fresh synovial biopsies obtained from four RA patients undergoing finger arthroplasty. All patients fulfilled the 1987 American Rheumatism Association criteria for RA [29]. The mean age of the patients was 67.4 ± 3.2 years (range 53–81 years). The mean disease duration was 8.7 ± 2.3 years. At the time of surgery, the disease activity score (DAS 28) was greater than 3.2. These activities were approved by local institutional review boards, and all subjects gave written informed consent. Synovia were minced and digested with 1.5 mg/mL collagenase-dispase for 3–4 h at 37 °C as previously described [30]. After centrifugation, cells were resuspended in DMEM supplemented with 10% FCS, 4.5 g/L D-glucose, 25 mM Hepes, 100 U/mL penicillin, and 100 μg/mL streptomycin (Gibco BRL) in a humidified atmosphere containing 5% (*v*/*v*) CO_2_ at 37 °C. After 48 h, nonadherent cells were removed. Adherent cells (macrophage-like and FLS) were cultured in complete medium, and, at confluence, cells were trypsinized and only the FLS were passed. These cells were used between passages 4 and 8, when they morphologically resembled FLS after an indirect immunofluorescence study (see Culture of human RA FLS). RA FLS were cultured 45–60 days before experimentation. This delay allowed for the elimination of all possible interactions resulting from any preoperative treatment (with nonsteroidal anti-inflammatory drugs, analgesics, disease-modifying antirheumatic drugs, or steroids).

### 2.3. Culture of Human RA FLS and Treatment

Between passages 4 and 8, RA FLS were trypsinized. Cell count and viability were determined, and cells were plated in culture plates or flasks (Falcon, Oxnard, CA, USA). Viability, measured by trypan blue dye exclusion [31] at the start and the end of culture, was always greater than 95%. FLS (10^5^) from RA patients were used for an indirect immunofluorescence study [32]. The following monoclonal antibodies were used: 5B5 (anti-prolyl hydroxylase) for fibroblasts at a 1/50 dilution (Dako, Burlingame, CA, USA), JC/70A (anti-CD31), for endothelial cells at 1/50 (Dako), and RMO52 (anti-CD14) for macrophages at 1/50 (Immunotech). The negative control was a mouse antibody of the same isotype (Immunotech). Incubations were performed at room temperature for 30 min. Binding of monoclonal antibodies was visualized using fluorescein (DTAF)-conjugated goat anti-mouse antibody (Immunotech) at a 1/50 dilution.

### 2.4. Antiproliferative Assays

RA FLS cells were trypsinized in fresh DMEM culture medium. Homogeneous solutions were prepared in 10 mL of medium with 700,000 cells. In a 96-well plate, 100 µL of the solution (7000 cells per well) was poured and the cells were incubated for 24 h at 37 °C and 5% CO_2_. Subsequently, 100 µL of PS solution in increasing concentration was poured per row in the plate and incubated for 24 h in the same conditions. The compounds were dissolved in DMSO (1 mM) just before use and then added to the culture medium in the desired concentrations. The concentration of DMSO in the cell medium was never more than 0.05%. After incubation, the medium was removed and 100 µL of complete medium without red phenol was added per well. At that point, irradiation was carried out using a red-light source, CureLight^®^, PhotoCure ASA at 630 nm, at a dose of 40 mW/cm^2^ for 30 min. After the irradiation, the wells plates were put in the incubator for 18 h. After this time, 10 µL of MTT solution (5 g/L) was added and the plates were again placed inside the incubator for 4 h. Next, the media was removed and 200 µL of DMSO was added per well, stirring the plate softly for 3 min. Absorbance after the MTT assay was measured at 540 nm by a Dynex Triad Multi Mode Microplate Reader, Dynex Technologies. The assays were executed in triplicate. Cytotoxicity evaluations in the dark were carried out by repeating this entire protocol without the irradiation dose.

### 2.5. Protein Extraction and Western-Blot Analysis

For total protein extraction, RA FLS were washed in PBS, and the total cell pool was centrifuged at 200 g for 5 min at 4 °C and homogenized in RIPA lysis buffer (50 mM HEPES, pH 7.5, 150 mM NaCl, 1% sodium deoxycholate, 1% NP-40, 0.1% SDS, and 20 mg/mL of aprotinin) containing protease inhibitors (CompleteTM Mini, Roche Diagnostics) according to the manufacturer’s instructions. Proteins (60 µg) were separated by electrophoresis on 10% SDS–PAGE gels and transferred to polyvinylidene fluoride (PVDF) membranes (Amersham Pharmacia Biotech, Saclay, France), which were then probed with a COX-2 human primary antibody (Cayman Chemical, Bertin Pharma, Montigny le Bretonneux, France). After incubation with a secondary antibody (Dako France S.A.S., Trappes, France), blots were developed using the ECL Plus Western Blotting Detection System (Amersham Pharmacia Biotech) and G: BOX system (Syngene, Ozyme, Saint Quentin en Yvelines, France). Membranes were then reblotted with human anti-β-actin (Sigma-Aldrich, Saint Quentin Fallavier, France) used as a loading control.

### 2.6. Assay of COX-2 Activity

RA FLS were maintained in DMEM supplemented with 10% (*v*/*v*) FCS, 4.5 g/L D-glucose, 100 U/mL penicillin, and 100 μg/mL streptomycin. The cells were grown in a humidified incubator at 37 °C and 5% CO_2_. Next, 2.10^6^ RA FLS cells were seeded in a 25 cm^2^ flask and incubated for 24 h. Then, the IC_50_ of each PS was added and the cells were incubated for 24 h. The medium was removed and a medium without red phenol was added. Immediately, cells were irradiated under the same conditions expressed in the MTT assays and incubated for 18 h. The non-irradiated cells were kept in the incubator. After this, LPS (1 µg/mL) was added to the medium of both irradiated and non-irradiated cells, and the cells were incubated for 4 h. Cells were trypsinized and the culture medium supernatant was isolated. The PGE_2_ levels were quantified in the culture media supernatants from treated and control cells by enzyme immunoassay using an ELISA Kit (Cayman Chemical) [33]. The results were expressed as the average of three independent experiments. 

### 2.7. Assay of IL-1β Production

The IL-1β levels were quantified in the culture media supernatants, isolated by the same protocol described for PGE_2_, from treated and control cells by ELISA Kit (Thermo Fisher Scientific). The results were expressed as the average of three independent experiments. 

### 2.8. Statistical Analysis

All quantitative results are expressed as the mean ± 3 standard deviations (SEM) of separate experiments using Excel (Microsoft Office, Version 2019). Statistical significance was evaluated by the two-tailed unpaired Student’s *t*-test, *p*-value < 0.001 (***).

## 3. Results and Discussion

### 3.1. Phototoxicity Tests

Although it is the first time that these PS–metallacage systems have been tested to treat RA using PDT, two of the fifteen systems described here (Figure 3) have already been tested in cancer (HeLa, Me300, A2780, A2780cisR, and A549) [21]. Specifically, the prismatic metallacage **M1** and the cubic **M4**, both with **G1** in their internal cavity. In cancer cells, a total absence of cytotoxicity was demonstrated prior to cell internalization. Once inside the cells, the PS is released from the cage and can be irradiated giving rise to photocytotoxicity. Two mechanisms have been suggested to explain the releasing of the PS from the metallacage: (i) from a partial or total rupture of the cage; or (ii) through an aperture [21,22]. Furthermore, intracellular ruthenium contents [22] and fluorescence studies [21] have confirmed the ability of these metallacages to cross cell membranes. Fluorescence studies also reveal that, once inside the cell and after the PS leaves the cavity of the metallacage, both are positioned in different cellular areas, which did not include the nucleus.

In this work, we wanted to demonstrate the efficacy and potential of these systems in another pathology, RA, looking for a treatment that is fairly non-invasive. In addition, we have synthesized cages with structural variations to evaluate how the different elements of the metallacage influence its PDT effect (Figure 3). Moreover, we have evaluated new PSs, such as **G2**, **G3,** and **G4**, in addition to **G1** which have been evaluated to treat RA by PDT.

First, these metallacages can be differentiated by their two main elements: the panel ligand and the dinuclear ruthenium clips. The panel ligand is a flat organic compound with three or four pyridine substituents, which give rise to prismatic or cubic cages, respectively. In this work, we used 2,4,6-tri(pyridin-4-yl)-1,3,5-triazine, 1,3,5-tris{2-(pyridin-4-yl)vinyl}benzene, or 1,2,4,5-tetrakis{2-(pyridine-4-yl)vinyl} benzene. Dinuclear arene ruthenium(II) complexes are the edges of the cage, whose two metal atoms are linked by 2,5-dioxydo-1,4-benzoquinonato, 5,8-dioxydo-1,4-naphthoquinonato, or 6,11-dioxydo-5,12-naphthacenedionato ligands (Figure 3).

The results of the photocytotoxicity tests after PDT in RA FLS were excellent (Table 1). MTT assays showed 50% inhibition concentrations (IC_50_) lower than those seen in cancer cells [21]. The latter was to be expected, since RA FLS are primary cells and their growth is not accelerated, unlike cancer cells. As we anticipated, the structural variation in the cages gave rise to significant differences in the PDT effect.

First, we have observed that when the size of the panel ligand is bigger, the photocytotoxicity is higher. For example, the structures of cages **M2**, **M4,** and **M6** differ only by the panel ligand, 2,4,6-tri(pyridin-4-yl)-1,3,5-triazine, 1,3,5-tris{2-(pyridin-4-yl)vinyl}benzene, and 1,2,4,5-tetrakis{2-(pyridine-4-yl)vinyl} benzene, respectively. When the IC_50_ values obtained with porphine as the PS are compared (entries 2, 4, and 6 in Table 1), we observed that cage **M2**, with the smallest panel, needed a higher concentration than **M4** and **M6** (triple when compared to **M6**). This difference becomes more evident if we compare cage **M1** and **M5**, which have panels 2,4,6-tri(pyridin-4-yl)-1,3,5-triazine and 1,3,5-tris{2-(pyridin-4-yl)vinyl}benzene, respectively. With **G1** as the PS, the IC_50_ of **M1** is six times higher than that observed in **M5** (entries 1 and 5 in Table 1). This coincides with what has been reported in cancer cells [21]. A larger panel gives rise to larger apertures that facilitate the release of the PS once inside the cell, producing more ROS and, subsequently, more photocytotoxicity. This result is consistent with the other three PSs tested (Table 1).

The second of the structural elements of the cages that we can modify, the dinuclear ruthenium clip, also showed significant differences, as we expected. When the volume of the ruthenium complex is bulkier, we observed that the IC_50_ is lower, which translates into a better PDT effect. For instance, cages **M1**, **M2**, and **M3** contain the same panel ligand and differ only in the dinuclear ruthenium “edges”, being 2,5-dioxydo-1,4-benzoquinonato, 5,8-dioxydo-1,4-naphthoquinonato, and 6,11-dioxydo-5,12-naphthacenedionato respectively. With **G1** as the PS, the result obtained with **M3** was four times lower than the IC_50_ obtained in **M1** (entries 1 and 3 in Table 1), while the IC_50_ of **M2** (entry 2 in Table 1) shows an intermediate value. These results are consistent with the structure of the metallacages, which suggest the release of the PS through an aperture [22]. Indeed, when the metallacage is smaller, the host–guest system is stabilized, making it difficult for the PS to escape, which translates into lower ROS production and a lower PDT effect. The same result, although in a lesser proportion, is observed with the other PSs tested (Table 1).

**Figure 4:**
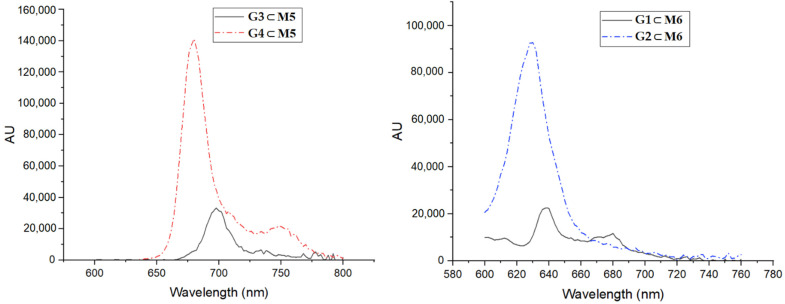
Emission spectra of **M5** with **G3** or **G4** (**left**) and **M6** with **G1** or **G2** (**right**), in DMSO at 25 °C (10 nM concentration).

Finally, the four PSs tested have shown significant differences. First, it is worth noting the presence or absence of a metal in the center of the tetrapyrrole. In all cases, using the same cage, the PSs without a metal showed a better PDT effect (Table 1). The cause of this result is directly related to fluorescence [34]. When the PS is irradiated, part of the energy is absorbed and the PS reaches the excited singlet state. The PS can then return to the minimum energy state by releasing that energy, producing fluorescence, or the energy can pass through an intermediate excited triplet state. From this last state, the PS can return to the ground state, giving rise to phosphorescence, or interact with O_2_ to give rise to singlet oxygen and, in turn, ROS [12,13]. Therefore, since the derivate with Zn and Mg give rise to higher fluorescence (Figure 4), lower ROS production would be expected than their equivalents without metal. This corroborates the obtained results, that is, the presence of Mg or Zn favor fluorescence and therefore reduce ROS production and PDT efficiency. Calculating the quantum yields (Table 1), we observed the same result as expected, that is, higher quantum yield equates to less of a PDT effect.

Regarding the differences between porphine (**G1**) and phthalocyanine (**G3**), the results show that **G1** works better as a PS than **G3** when carried in the same metallacage (Table 1). However, the IC_50_ for **G3** is still excellent, with both showing great potential as PSs. Surprisingly, one of the results was unexpected. When **G3** or **G4** is transported by the cubic metallacage (**M4**), no effect on RA FLS is observed (Table 1), even at the highest concentration tested. This also suggests a stronger binding affinity between the host and the guest, thus supporting that the PS is released through an aperture, rather than having a breakage of the metallacage [22].

**Figure 5:**
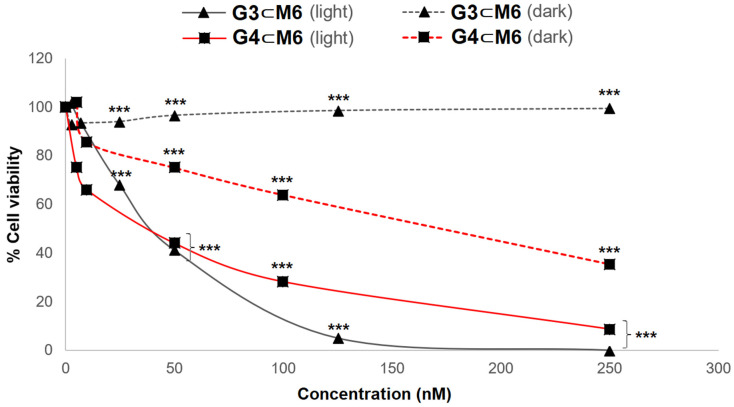
MTT assays of **G3**⊂**M6** (black) and **G4**⊂**M6** (red), in the dark (dashed line) and after irradiation (solid line). Statistical significance determined by the two-tailed unpaired Student’s *t*-test, *p*-value < 0.001 (***).

Another excellent result is the total absence of cytotoxicity in the dark for all compounds, except for those with **G4** in their cavity, which show dark toxicity (Figure 5). Therefore, this result suggests that **G4** is not a good PS, although it is something we could have anticipated since other zinc tetrapyrrole derivatives have already been reported to show toxicity in the dark [35,36]. Another intriguing result comes from the metallated photosensitizers (**G3** and **G4**) encapsulated in the 1,2,4,5-tetrakis{2-(pyridine-4-yl)vinyl} benzene derivative (**M4**) (entries 10 and 13, Table 1). In both systems (**G3**⊂**M4** and **G4**⊂**M4**), no phototoxicity and no toxicity is observed, suggesting the absence of a photo-response from the photosensitizers in these particular cases. When compared to the other **G**⊂**M** systems, the most plausible explanation is that the presence of Mg or Zn in the core of the PS generates a stronger interaction between the host and the guest, thus shielding the PS and blocking their release. 

### 3.2. Inflammatory Evaluation

The synovial membrane encapsulates the joint, providing structural support, lubricating the tissues, and providing nutrients to the cartilage. FLS are part of the inner lining layer of the synovial membrane. One of the main functions of FLS is the production of cytokines [37]. One of the cytokines involved in the inflammatory response is the interleukin (IL) family. IL-1 can express cyclooxygenase-2 (COX-2), an enzyme that acts as a catalyst in the production of prostaglandin E_2_ (PGE_2_) from arachidonic acid [38,39,40,41]. PGE_2_ causes vasodilation in the synovial tissue, leading to inflammation in the area [42]. To evaluate in RA FLS the in vitro inflammatory activity after PDT, we decided to measure the production of PGE_2_ and IL-1β in the supernatant, in addition to the expression of COX-2 in both the irradiated and non-irradiated treated cells.

**Figure 6:**
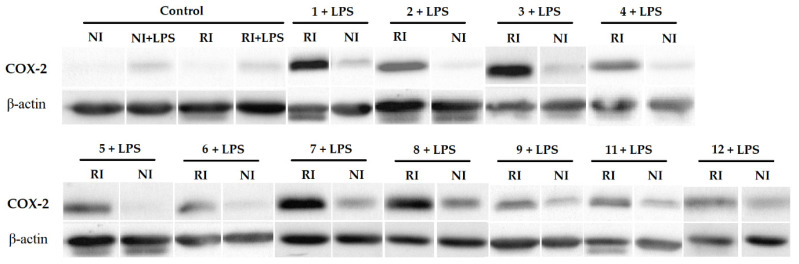
Effects of the systems tested on COX-2 expression after PDT. The numbers correspond to the entries in Table 1. Cells (2 × 10^6^) were cultured in DMEM medium (FBS 10%, L-glutamine 1%, penicillin 100 U/mL, Streptomycin 100 µg/mL) for 24 h and treated with the corresponding system **G**⊂**M**. After 24 h, the medium was replaced by a DMEM medium without red phenol, and then irradiated (RI) or not (NI) by 630 nm irradiation (40 mW/cm^2^, 30 min). After 18 h, LPS (1 µg/mL) was added to the medium to stimulate the expression of COX-2, and 4 h later the trypsination was carried out. COX-2 expression was determined by Western Blot and β-actin was used as a protein loading control. All experiments were done in triplicate.

The determination of COX-2 expression reveals that treated RA FLS with our systems by PDT generates an overexpression of this enzyme (Figure 6), when the cells were irradiated. This result was expected, since multiple examples of COX-2 overexpression after PDT have been reported. For instance, other porphyrin-based PSs such as PpIX-polyamine [43] or Photofrin [44] increased COX-2 expression. Additionally, this not only happens with PSs based on porphyrins, but also with other PSs used in PDT [45,46]. It should be noted that most of the systems with a lower IC_50_ (Table 1) show less intensity in the COX-2 expression band (Figure 6). For instance, the compounds that obtained the lowest IC_50_, entries 4, 5, 6, 11, and 12 (Table 1), showed a COX-2 band with the lowest intensity.

As expected, an overexpression of COX-2 generates a greater production of PGE_2_ [43], which may lead to an increase in inflammation. That is what we see in the results obtained in the determination of PGE_2_ (Table 2). As with COX-2, it can be seen that when the IC_50_ is lower, the production of PGE_2_ is also lower, which again points out that reducing the required concentration of PS could reduce the adverse effects of PDT. However, it is possible to minimize the expression of COX-2 and, consequently, the production of PGE_2_ by using a COX-2 inhibitor, such as NS-398 during PDT treatment [43,47].

On the other hand, IL-1β is known to be a pro-inflammatory cytokine that leads to the expression of COX-2, among other functions [48]. Since our experiments showed an overexpression of COX-2 and the production of PGE_2_, we anticipated the presence of this cytokine as a response to the PDT treatment. Unexpectedly, the determination of IL-1β indicates that its presence after PDT is insignificant (Table 2). It is even below the standard of lower concentration and their values were not significantly different from the control samples. This indicates that, in vitro, when RA FLS are treated with our systems by PDT, IL-1β is not generating more COX-2 than what is already present in the cells, so it is not involved in the detected overexpression. However, also in synovial tissues, other cases have been reported in which IL-1β was not involved in the overexpression of COX-2 [49,50,51], indicating that other cytokines like IL-6 or IL-8 were responsible [51].

## 4. Conclusions

A series of photosensitizers (**G**) encapsulated in arene ruthenium metallacages (**M**) have been synthesized and characterized. The PDT effect of these host–guest systems (**G**⊂**M**) has been evaluated on fibroblast-like synoviocyte cells (FLS). With the exception of the zinc phthalocyanine derivatives (**G4**⊂**M5** and **G4**⊂**M6**), all **G**⊂**M** compounds show no toxicity in the dark at the highest concentration tested (1.5 μM). When under light, the most photoactive compounds appear to be those with the largest cavity and the smallest guest, suggesting that the release of the photosensitizers from the host occurs without any breakage of the metallacage. However, when **G4** is encapsulated in the metallacages built with 1,3,5-tris{2-(pyridin-4-yl)vinyl} benzene panels (**M5** and **M6**), the difference between phototoxicity and toxicity is limited. On the other hand, when the metallated photosensitizers (**G3** and **G4**) are encapsulated in the 1,2,4,5-tetrakis{2-(pyridine-4-yl)vinyl} benzene derivative (**M4**), no phototoxicity is observed, suggesting a strong interaction between the host and guest, which shields the photosensitizer. Nevertheless, in all systems, PDT gives rise to the overexpression of COX-2 and PGE_2_. However, we have also observed that when a lower concentration of the drug is used, this overexpression is significantly reduced. Surprisingly, IL-1β does not seem to be involved in this COX-2 overexpression, despite being previously reported. This indicates that other cytokines are responsible for this overexpression of COX-2. With a few exceptions, all systems show encouraging results, and further in vitro investigations should be performed and other host–guest systems evaluated in order to validate our strategy; however, we think our results show an interesting method for the treatment of RA by PDT. This work, added to those already reported in the last three decades, both in vitro and in vivo, show the inherent potential that PDT could have in the treatment of RA.

## Figures and Tables

**Table 1:** Results of the MTT assays. Irradiation after 24 h of incubation with G ⊂ M, λ = 630 nm, 40 mW/cm^2^ for 30 min. IC_50_ values were calculated fitting the curve to a second degree polynomial ± 3 sigma deviations. The maximum concentration tested was 1.5 µM. Quantum yield (Φ_F_) was calculated using TPP as an internal standard in DMSO at 25 °C.

Entry	G ⊂ M	IC_50_ (nM) Light	IC_50_ (nM) Dark	ΦF (%)
**1**	**G1** ⊂ **M1**	211.7 ± 5.8	>1500	-
**2**	**G1** ⊂ **M2**	95.0 ± 5.9	>1500	-
**3**	**G1** ⊂ **M3**	53.6 ± 4.3	>1500	-
**4**	**G1** ⊂ **M4**	48.1 ± 9.7	>1500	-
**5**	**G1** ⊂ **M5**	35.4 ± 4.7	>1500	0.8
**6**	**G1** ⊂ **M6**	31.7 ± 6.6	>1500	1.1
**7**	**G2** ⊂ **M1**	302.6 ± 5.2	>1500	-
**8**	**G2** ⊂ **M4**	100.7 ± 5.8	>1500	-
**9**	**G2** ⊂ **M6**	91.8 ± 8.3	>1500	2.0
**10**	**G3** ⊂ **M4**	>1500	>1500	-
**11**	**G3** ⊂ **M5**	53.4 ± 4.5	>1500	0.11
**12**	**G3** ⊂ **M6**	47.4 ± 6.3	>1500	-
**13**	**G4** ⊂ **M4**	>1500	>1500	-
**14**	**G4** ⊂ **M5**	66.0 ± 2.6	103.8 ± 2.9	1.6
**15**	**G4** ⊂ **M6**	64.4 ± 4.4	163.8 ± 17.1	-

**Table 2:** PGE_2_ and IL-1β results. The assays were performed using the protocol provided by the ELISA kit in triplicate. The data were treated as explained in this protocol. The cells tested were treated by PDT with each of the indicated compounds as described in the experimental section. The control sample was treated exactly as the cells tested, that is, 18 h after the irradiation dose, 1 µg/mL of LPS was added to the medium and the cells were incubated for 4 h, then trypsinized and the cells and supernatant were isolated. The results are expressed by the average of three independent experiments. After testing the photocytotoxic activity, we chose the systems with the greatest potential to be used in PDT against RA and evaluated their inflammatory activity. Of these fifteen systems, we obviously ruled out those that did not work (cubic cage **M4** + phthalocyanines) and the systems that generated toxicity in the dark.

Entry	G ⊂ M	PGE_2_ (pg/mL)	IL-1β (pg/mL)
**Ctrl**	-	286.6 ± 0.1	1.8 ± 0.7
**1**	**G1** ⊂ **M1**	460.8 ± 4.3	2.3 ± 1.2
**2**	**G1** ⊂ **M2**	471.2 ± 3.4	1.9 ± 1.0
**3**	**G1** ⊂ **M3**	445.1 ± 4.7	2.8 ± 0.1
**4**	**G1** ⊂ **M4**	378.3 ± 14.2	3.2 ± 0.4
**5**	**G1** ⊂ **M5**	407.4 ± 14.5	2.1 ± 0.2
**6**	**G1** ⊂ **M6**	439.2 ± 10.1	1.6 ± 0.1
**7**	**G2** ⊂ **M1**	476.8 ± 3.4	1.9 ± 0.6
**8**	**G2** ⊂ **M4**	473.6 ± 7.5	1.4 ± 0.2
**9**	**G2** ⊂ **M6**	430.6 ± 1.4	2.2 ± 0.2
**10**	**G3** ⊂ **M5**	368.2 ± 26.5	2.4 ± 0.4
**11**	**G3** ⊂ **M6**	425.2 ± 2.7	0.1 ± 0.1

## Data Availability

Not applicable.

## Supplementary Materials

The following are available online at https://www.mdpi.com/article/10.3390/pharmaceutics13122104/s1, Figure S1: ^1^H NMR spectrum of **G1****⊂****M2** in CD_3_CN at 25 °C, Figure S2: ^13^C NMR spectrum of **G1**⊂**M2** in CD_3_CN at 25 °C, Figure S3: ^1^H-^1^H COSY NMR spectrum of **G1**⊂**M2** in CD_3_CN at 25 °C, Figure S4: DOSY NMR spectrum of **G1**⊂**M2** in CD_3_CN at 25 °C, Figure S5: ^1^H-^13^C HSQC NMR spectrum of **G1**⊂**M2** in CD_3_CN at 25 °C, Figure S6: ^1^H-^13^C HMQC NMR spectrum of **G1**⊂**M2** in CD_3_CN at 25 °C, Figure S7: ESI-MS spectrum of **G1**⊂**M2**, Figure S8: UV-vis absorbance spectrum of **G1**⊂**M2** (10 µM in DMSO), Figure S9: ATR FT-IR spectrum of **G1**⊂**M2**, Figure S10: 1H NMR spectrum of **G1****⊂****M3** in CD_3_CN at 25 °C, Figure S11: ^13^C NMR spectrum of **G1****⊂****M3** in CD_3_CN at 25 °C, Figure S12: ^1^H-^1^H COSY NMR spectrum of **G1****⊂****M3** in CD_3_CN at 25 °C, Figure S13: DOSY NMR spectrum of **G1****⊂****M3** in CD_3_CN at 25 °C, Figure S14: ^1^H-^13^C HSQC NMR spectrum of **G1****⊂****M3** in CD_3_CN at 25 °C, Figure S15: ^1^H-^13^C HMQC NMR spectrum of **G1****⊂****M3** in CD_3_CN at 25 °C, Figure S16: UV-vis absorbance spectrum of **G1****⊂****M3** (10 µM in DMSO), Figure S17: ATR FT-IR spectrum spectrum of **G1****⊂****M3**, Figure S18: ^1^H NMR spectrum of **G2****⊂****M1** in DMSO-d_6_ at 25 °C, Figure S19: ^13^C NMR spectrum of **G2****⊂****M1** in DMSO-d_6_ at 25 °C, Figure S20: ^1^H-^1^H COSY NMR spectrum of **G2****⊂****M1** in DMSO-d_6_ at 25 °C, Figure S21: DOSY NMR spectrum of **G2****⊂****M1** in DMSO-d_6_ at 25 °C, Figure S22: ^1^H-^13^C HSQC NMR spectrum of **G2****⊂****M1** in DMSO-d_6_ at 25 °C, Figure S23: ^1^H-^13^C HMQC NMR spectrum of **G2****⊂****M1** in DMSO-d_6_ at 25 °C, Figure S24: ESI-MS spectrum of **G2****⊂****M1**, Figure S25: UV-vis absorbance spectrum of **G2****⊂****M1** (10 µM in DMSO), Figure S26: ATR FT-IR spectrum spectrum of **G2****⊂****M1**, Figure S27: ^1^H NMR spectrum of **G2****⊂****M4** in CD_3_CN at 25 °C, Figure S28: ^13^C NMR spectrum of **G2****⊂****M4** in CD_3_CN at 25 °C, Figure S29: ^1^H-^1^H COSY NMR spectrum of **G2****⊂****M4** in CD_3_CN at 25 °C, Figure S30: DOSY NMR spectrum of **G2****⊂****M4** in CD_3_CN at 25 °C, Figure S31: ^1^H-^13^C HSQC NMR spectrum of **G2****⊂****M4** in CD_3_CN at 25 °C, Figure S32: ^1^H-^13^C HMQC NMR spectrum of **G2****⊂****M4** in CD_3_CN at 25 °C, Figure S33: ESI-MS spectrum of **G2****⊂****M4**, Figure S34: UV-vis absorbance spectrum of **G2****⊂****M4** (10 µM in DMSO), Figure S35: ATR FT-IR spectrum spectrum of **G2****⊂****M4**, Figure S36: ^1^H NMR spectrum of **G2****⊂****M6** in CD_3_CN at 25 °C, Figure S37: ^13^C NMR spectrum of **G2****⊂****M6** in CD_3_CN at 25 °C, Figure S38: ^1^H-^1^H COSY NMR spectrum of **G2****⊂****M6** in CD_3_CN at 25 °C, Figure S39: ^1^H-^13^C HSQC NMR spectrum of **G2****⊂****M6** in CD_3_CN at 25 °C, Figure S40: ^1^H-^13^C HMQC NMR spectrum of **G2****⊂****M6** in CD_3_CN at 25 °C, Figure S41: ESI-MS spectrum of **G2****⊂****M6**, Figure S42: UV-vis absorbance spectrum of **G2****⊂****M6** (10 µM in DMSO), Figure S43: ATR FT-IR spectrum spectrum of **G2****⊂****M6**.
