# Supplementary material for: Evaluation of Ruthenium-Based Assemblies as Carriers of Photosensitizers to Treat Rheumatoid Arthritis by Photodynamic Therapy
Source: Pharmaceutics. 2021 Dec 7;13(12):2104. doi: 10.3390/pharmaceutics13122104 (PMC8706357; doi:10.3390/pharmaceutics13122104)
Supplement: Supplementary file 1 [file pharmaceutics-13-02104-s001.zip › pharmaceutics-1467906-supplementary.pdf]

pharmaceutics

MDPI

Supplementary Materials: Evaluation of Ruthenium-Based Assemblies as Carriers of Photosensitizers to Treat Rheumatoid Arthritis by Photodynamic Therapy

INDEX

Characterization of compounds …………………………………..……….…….…….…...S1
1.1 Synthesis of G1⊂M2 ………………………………………………………….……....………S1
1.2 Synthesis of G1⊂M3 …………………………………………………………...…….…...S6
1.3 Synthesis of G2⊂M1 ……………………………………………...…………...…..……S10
1.4 Synthesis of G2⊂M4 ……………………………………………...…………...………S14
1.5 Synthesis of G2⊂M6 …………………………………………………………......…..……S18

1. Characterization of compounds

1.1 Synthesis of G1⊂M2

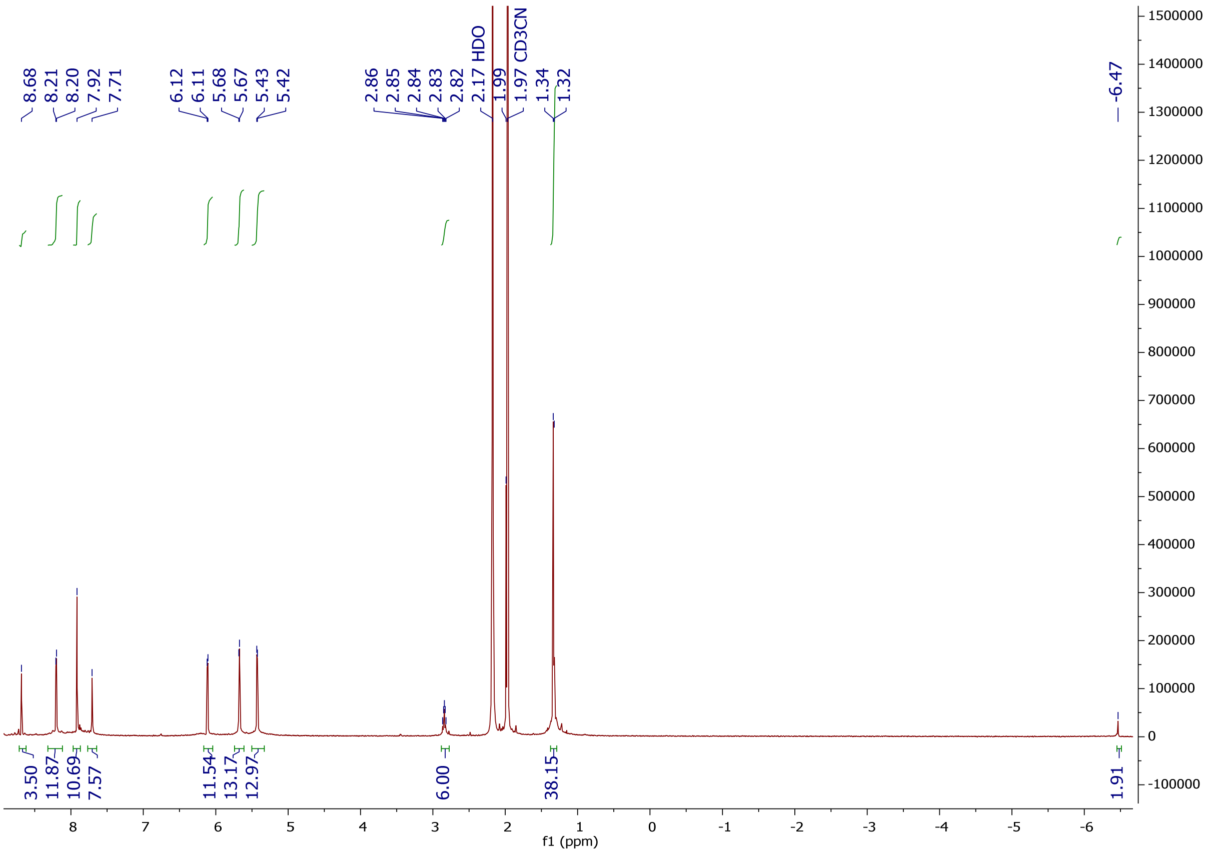

Figure S1. ^{1}H NMR spectrum of **G1⊂M2** in CD_3CN at 25 °C.

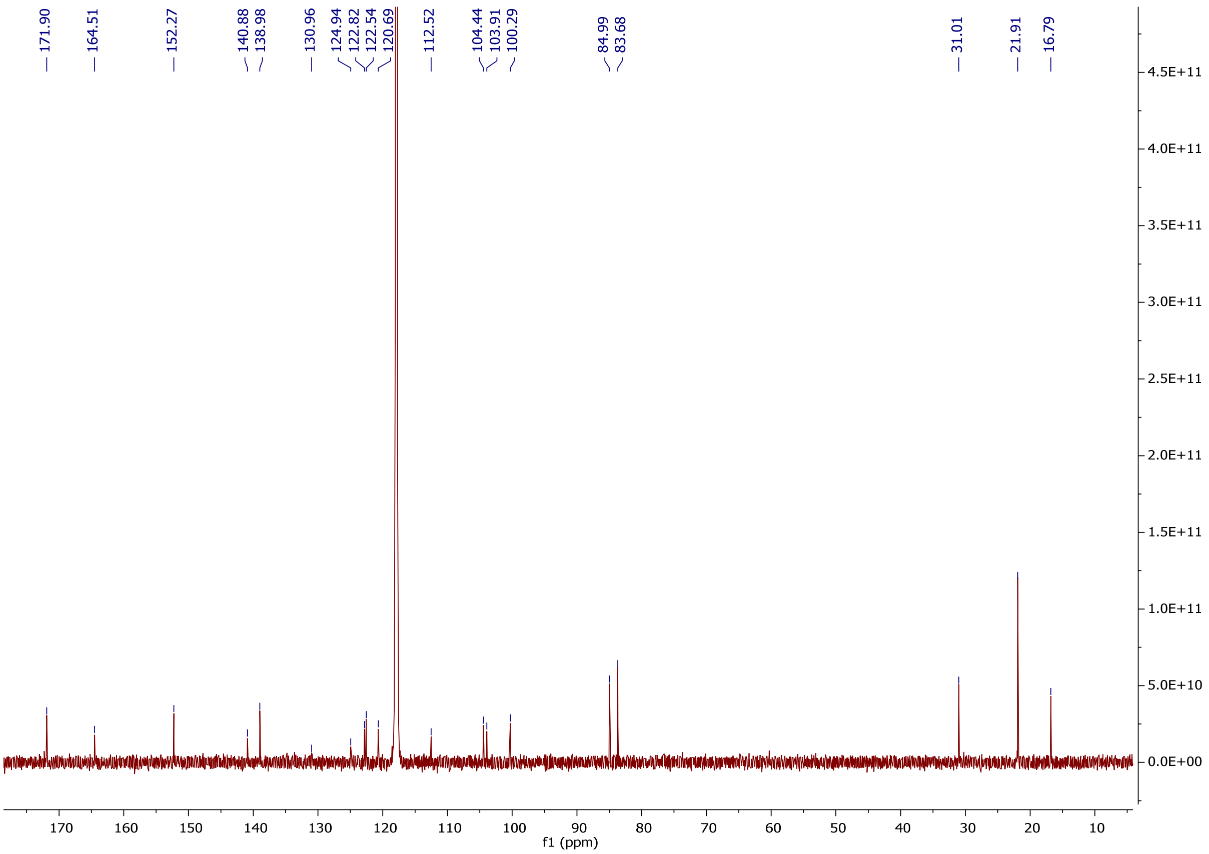

Figure S2. ^{13}C NMR spectrum of **G1⊂M2** in CD_3CN at 25 °C.

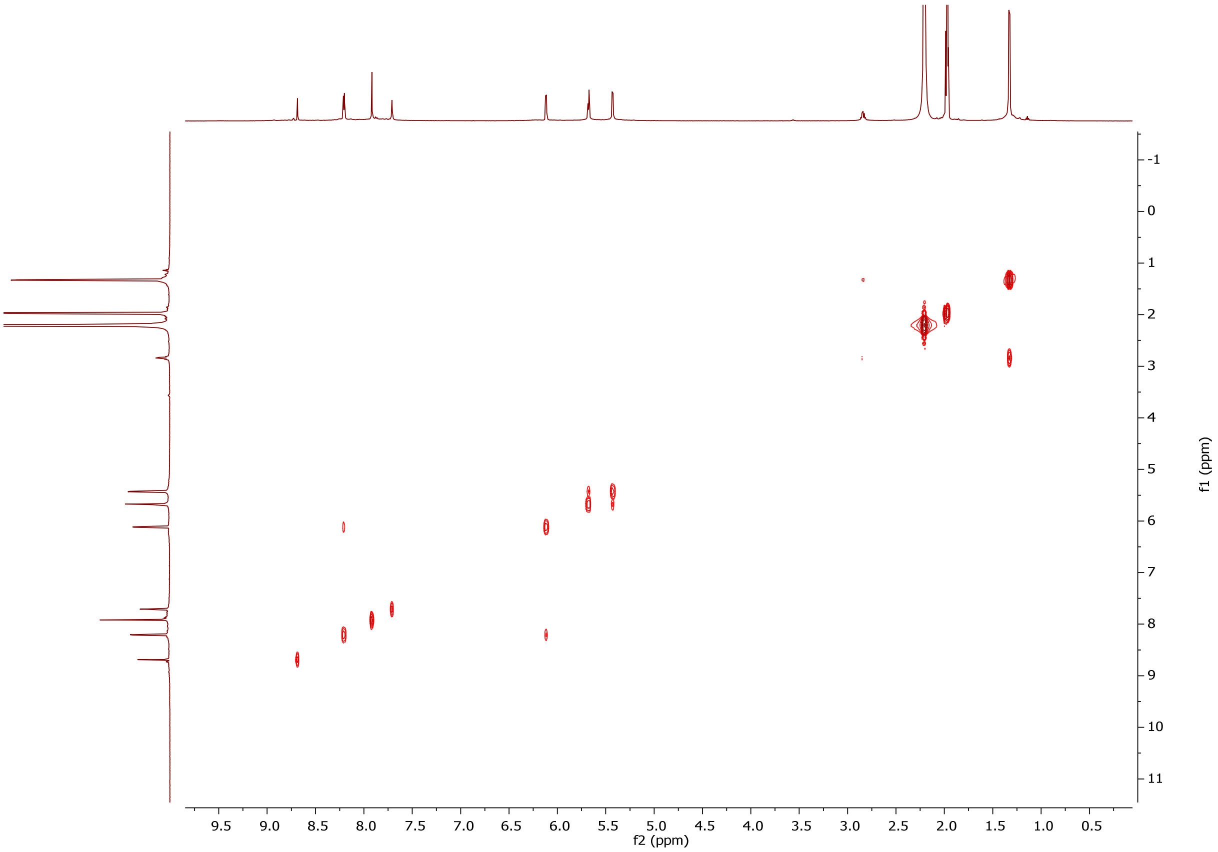

Figure S3. ^{1}H-^{1}H COSY NMR spectrum of **G1⊂M2** in CD_3CN at 25 °C.

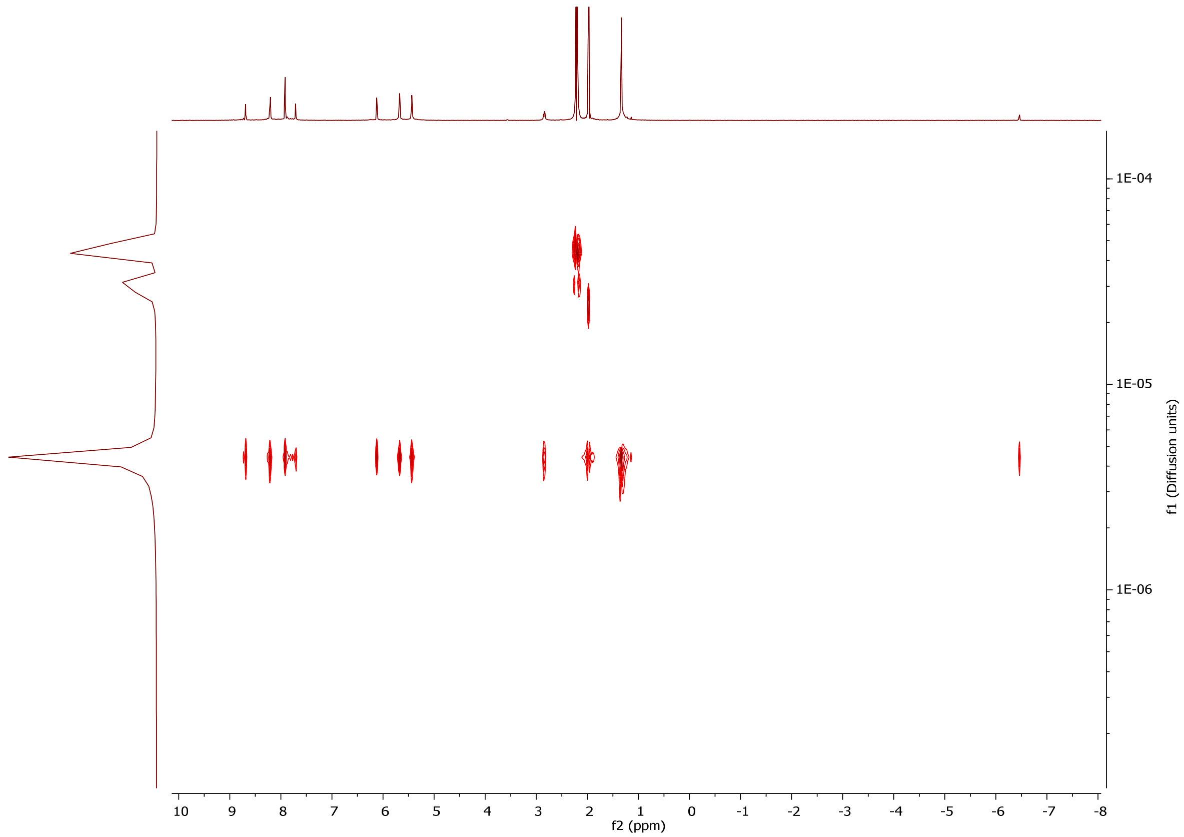

Figure S4. DOSY NMR spectrum of **G1⊂M2** in CD_3CN at 25 °C.

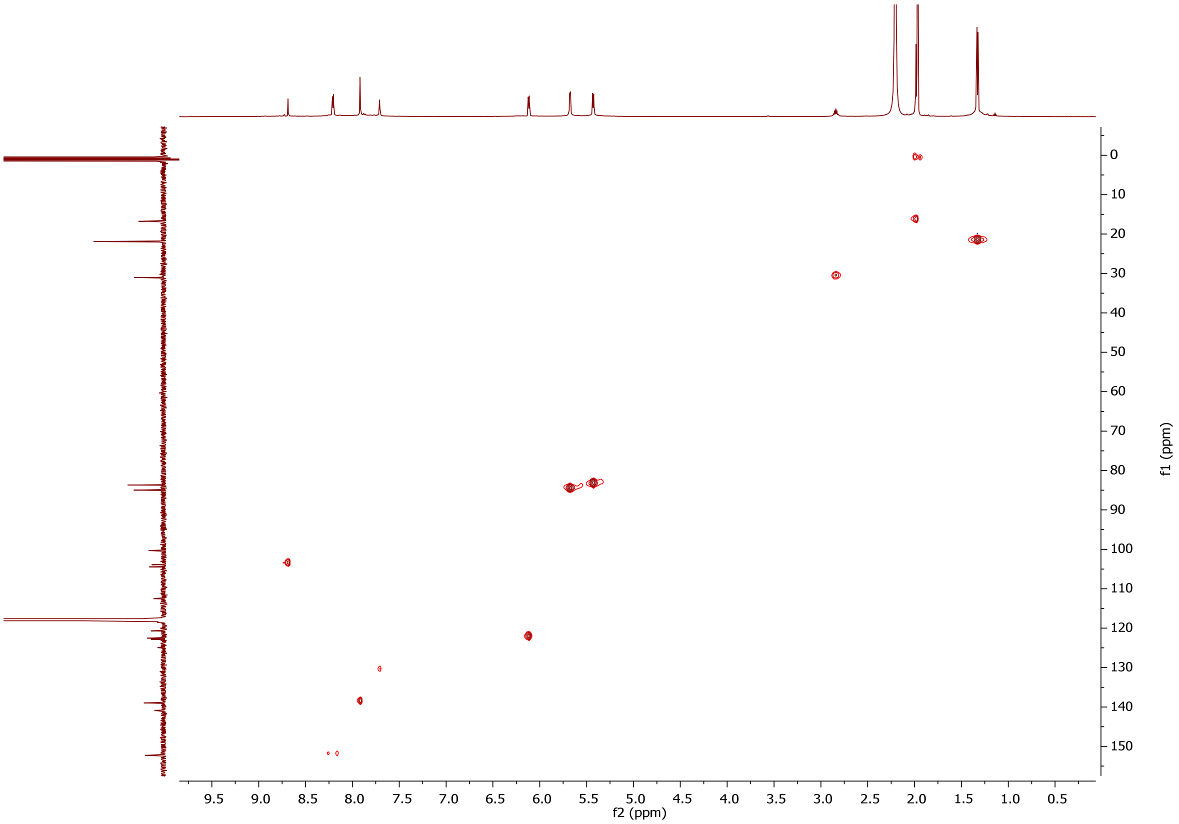

Figure S5. ^{1}H-^{13}C HSQC NMR spectrum of **G1⊂M2** in CD_3CN at 25 °C.

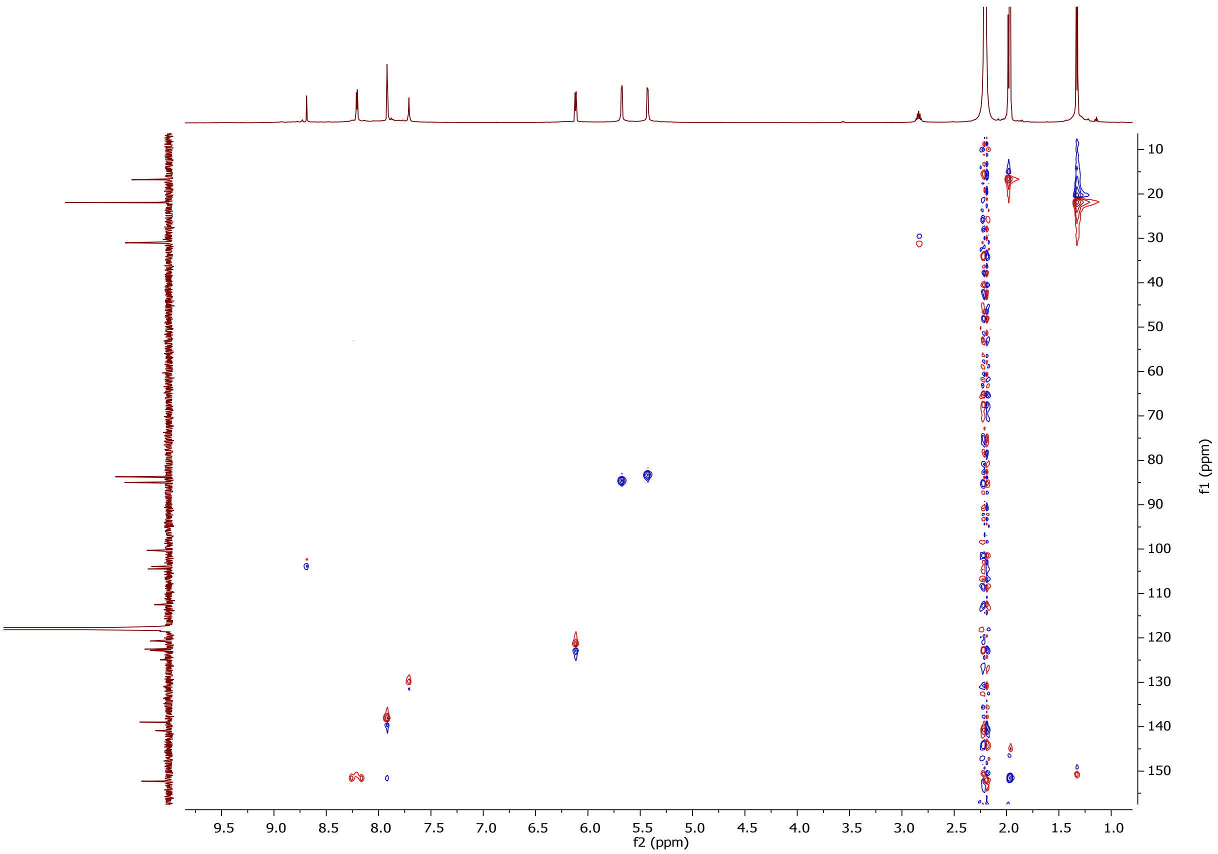

Figure S6. ^{1}H-^{13}C HMQC NMR spectrum of **G1⊂M2** in CD_3CN at 25 °C.

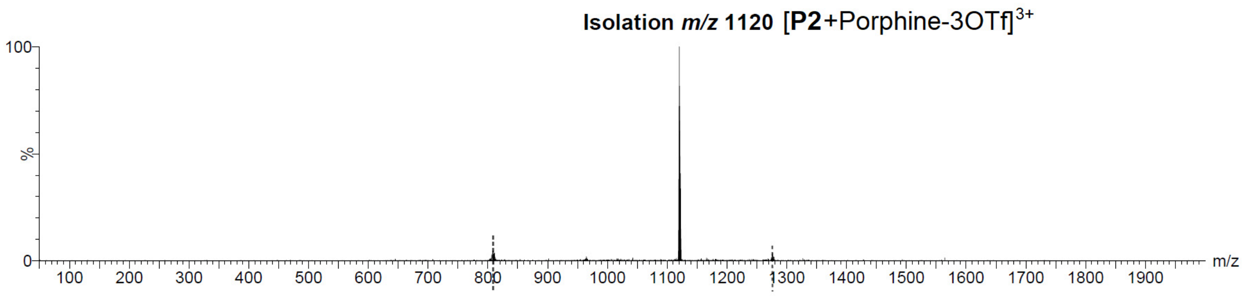

Figure S7. ESI-MS spectrum of **G1⊂M2**.

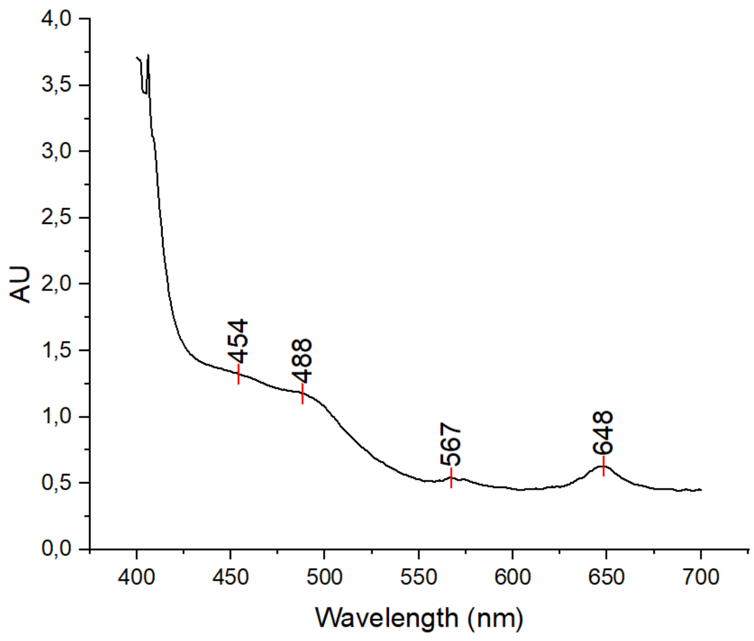

Figure S8. UV-vis absorbance spectrum of **G1⊂M2** (10 μM in DMSO).

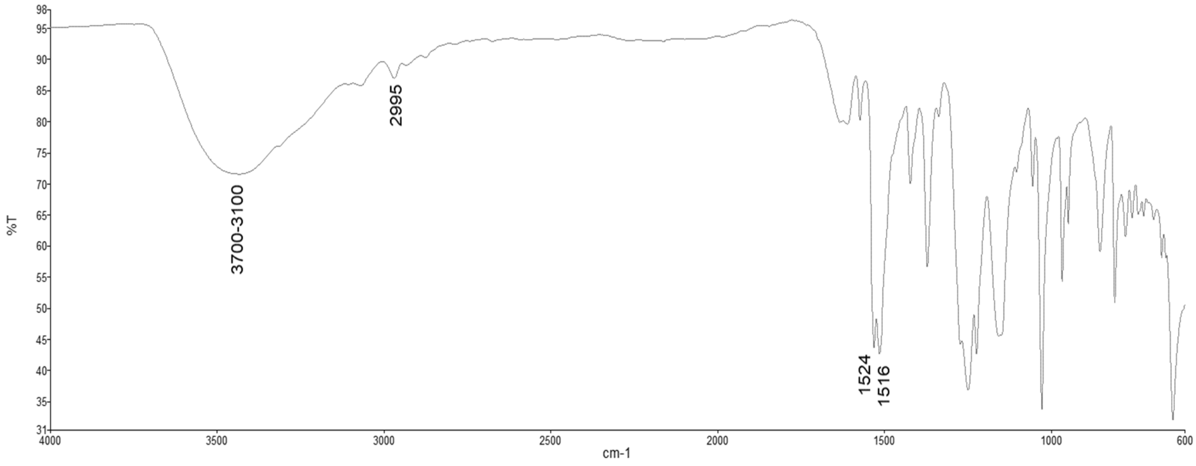

Figure S9. ATR FT-IR spectrum spectrum of **G1⊂M2**.

1.2 Synthesis of G1⊂M3

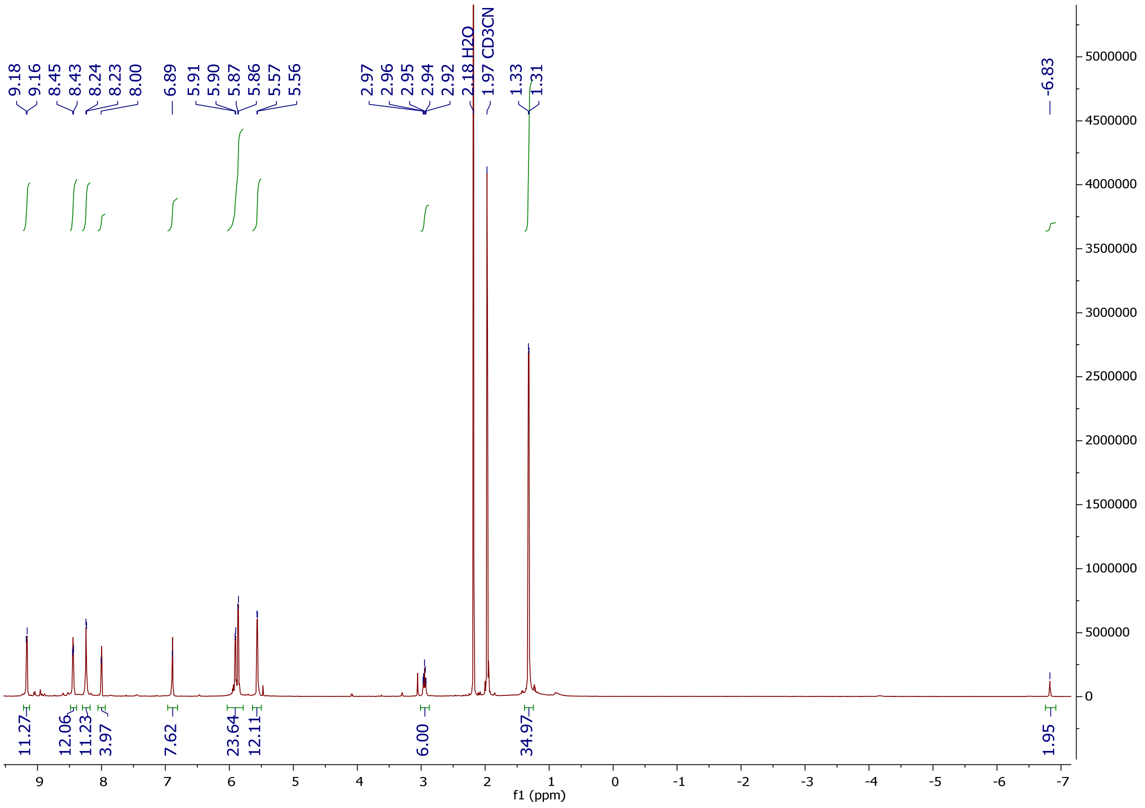

Figure S10. ^{1}H NMR spectrum of **G1⊂M3** in CD_3CN at 25 °C.

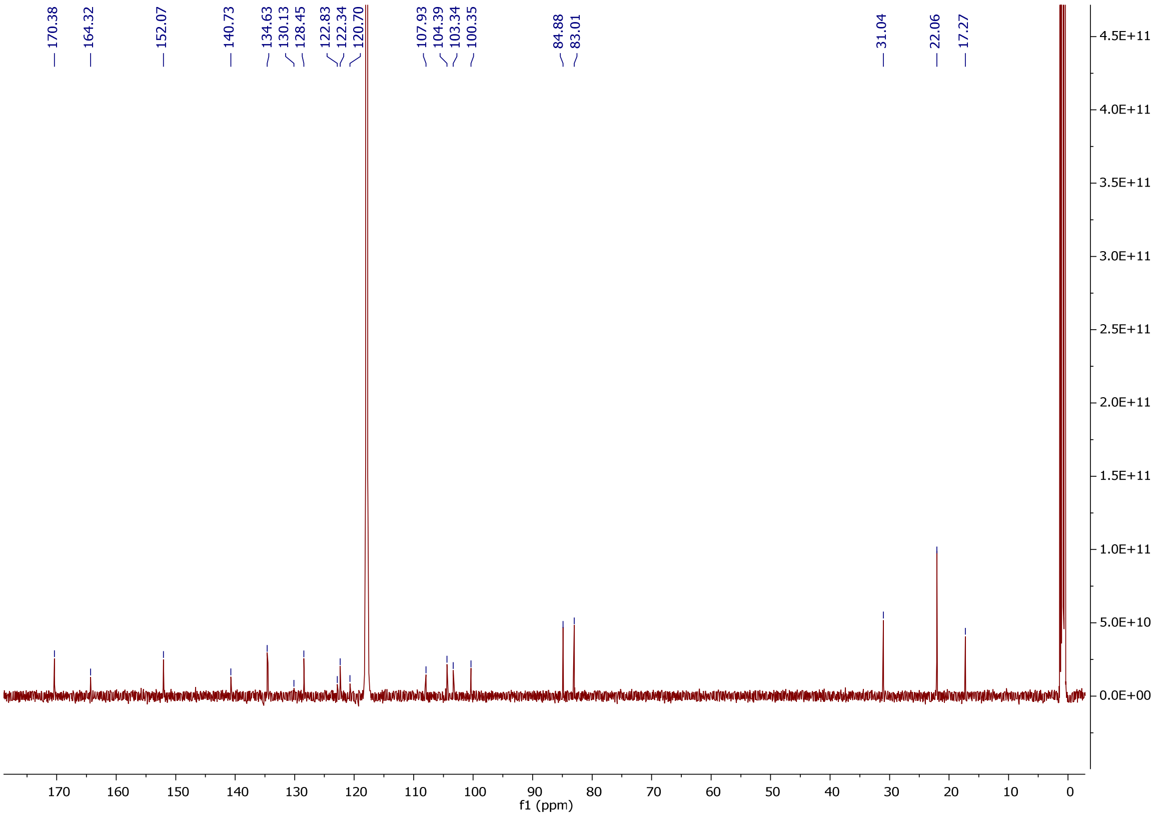

Figure S11. ^{13}C NMR spectrum of **G1⊂M3** in CD_3CN at 25 °C.

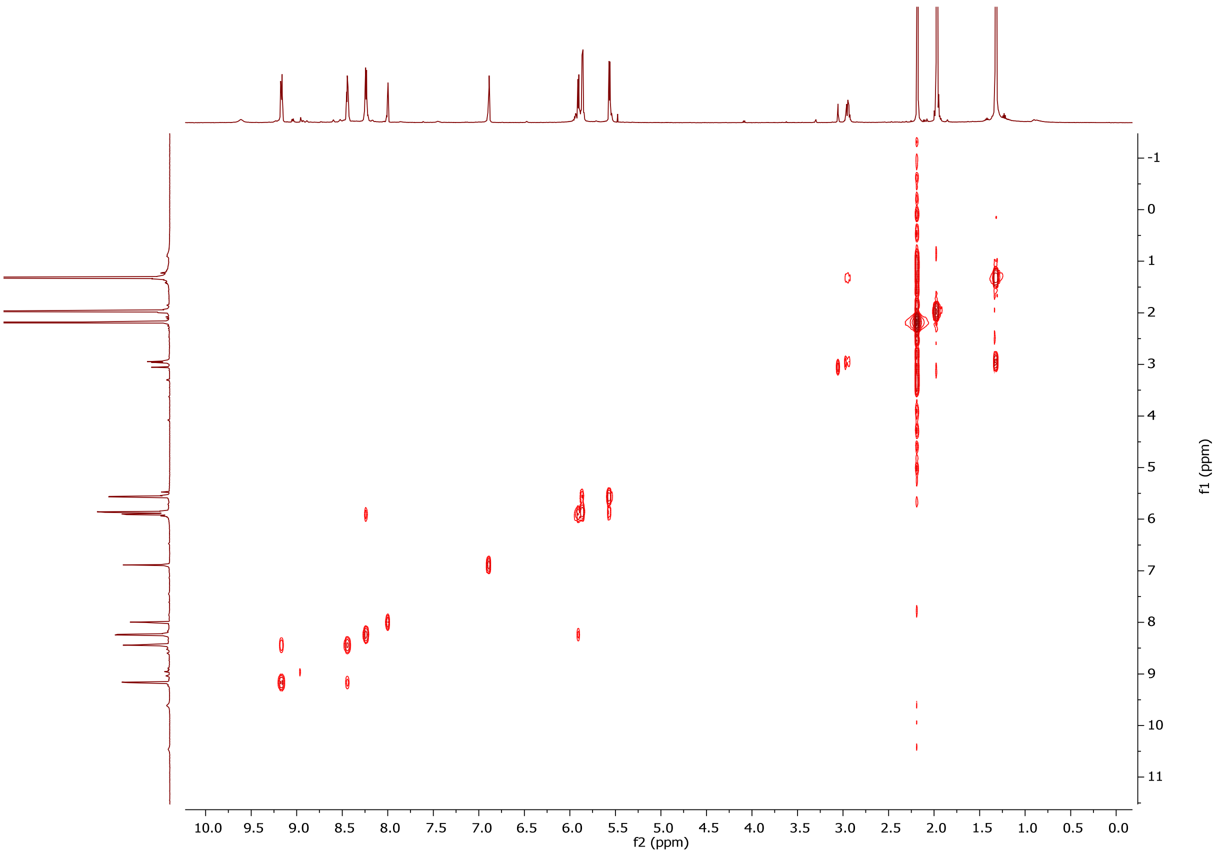

Figure S12. ^{1}H-^{1}H COSY NMR spectrum of **G1⊂M3** in CD_3CN at 25 °C.

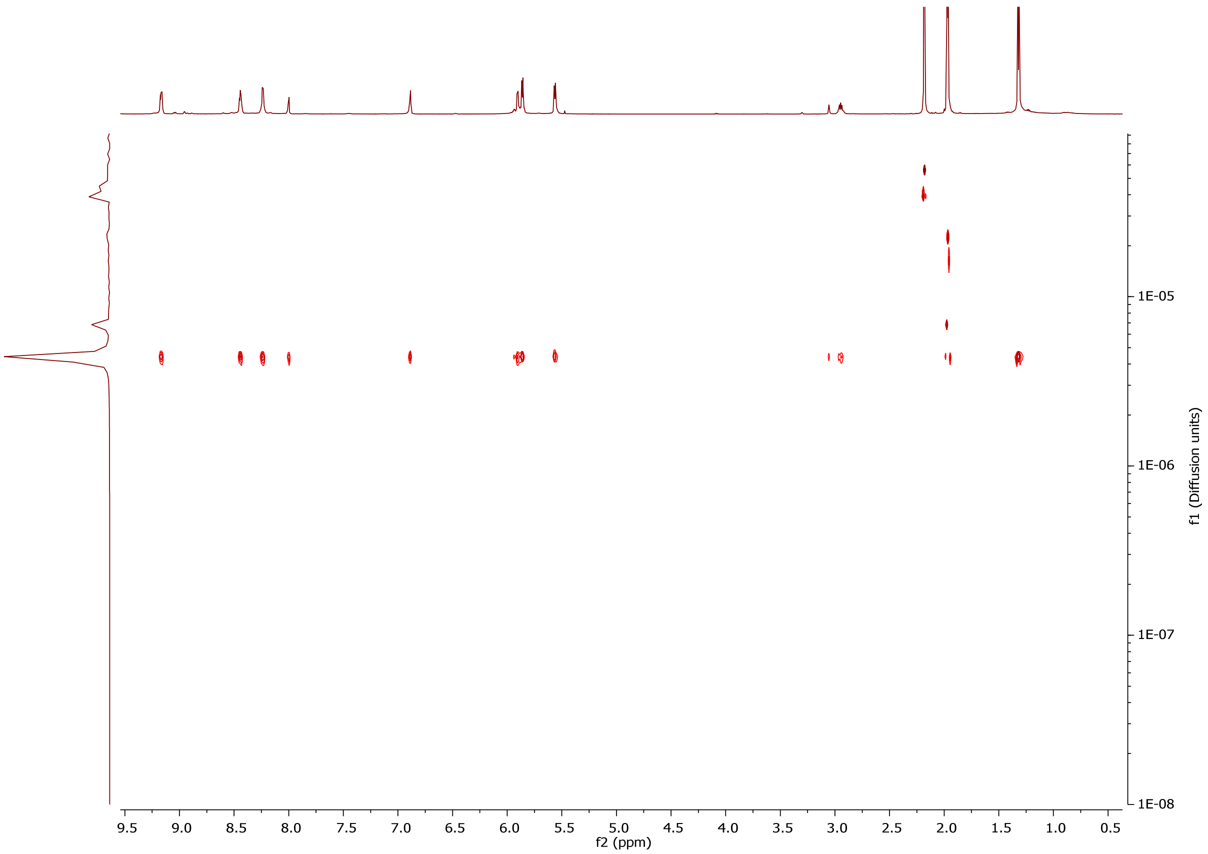

Figure S13. DOSY NMR spectrum of **G1⊂M3** in CD_3CN at 25 °C.

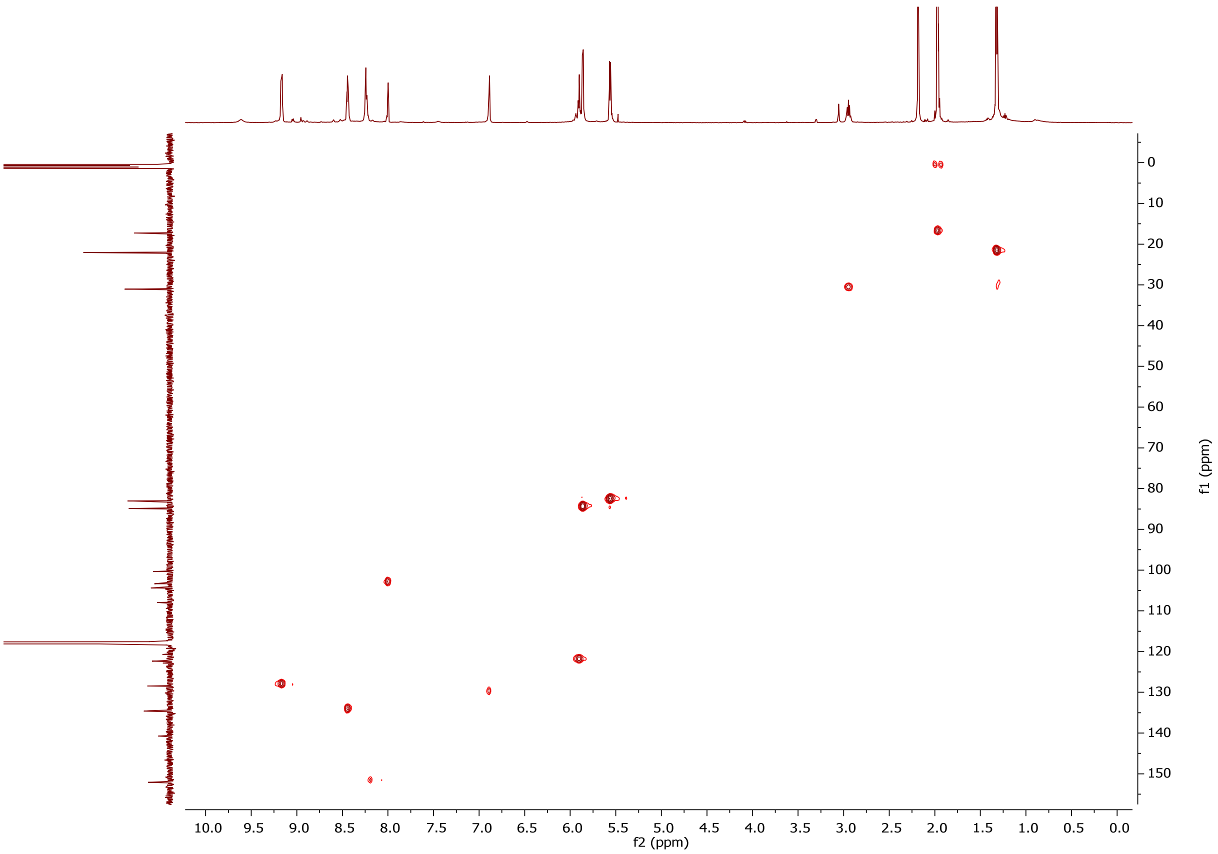

Figure S14. ^1H-^{13}C HSQC NMR spectrum of **G1⊂M3** in CD_3CN at 25 °C.

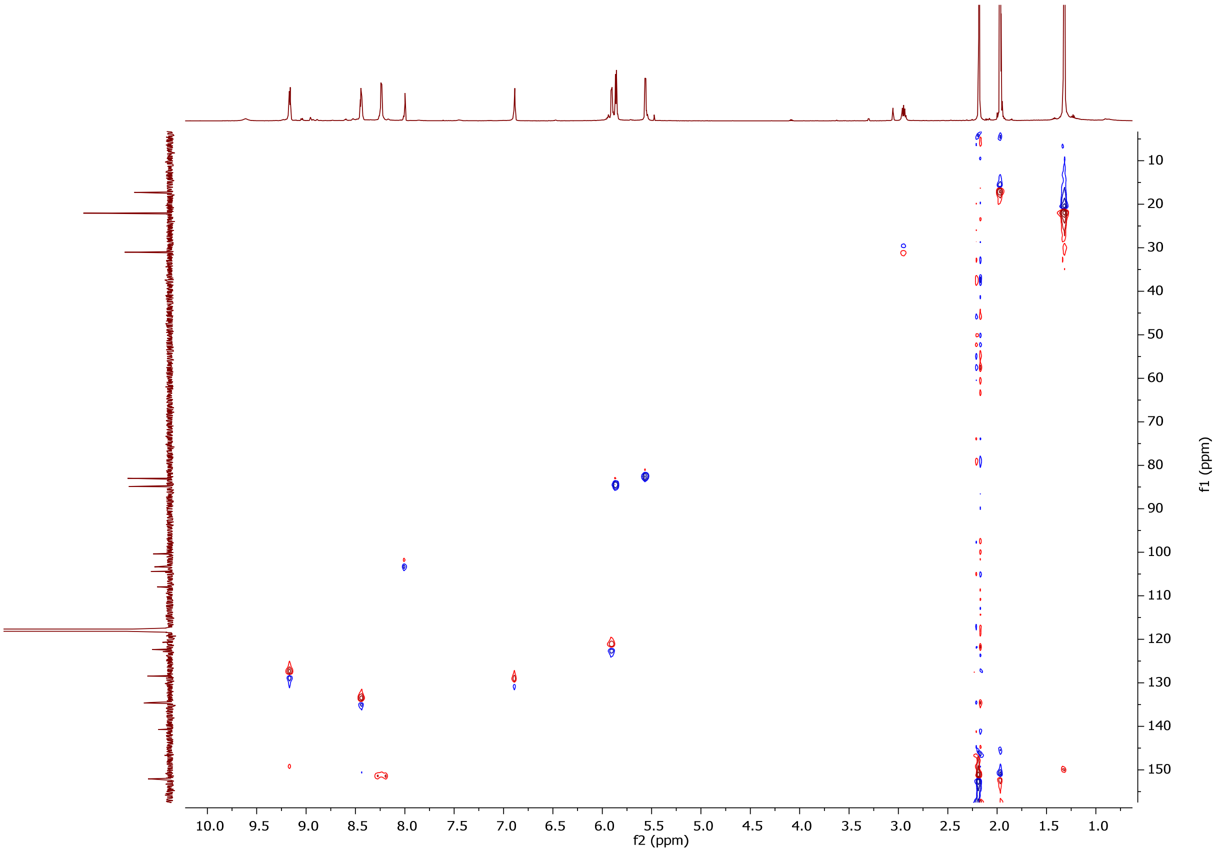

Figure S15. ^1H-^{13}C HMQC NMR spectrum of **G1⊂M3** in CD_3CN at 25 °C.

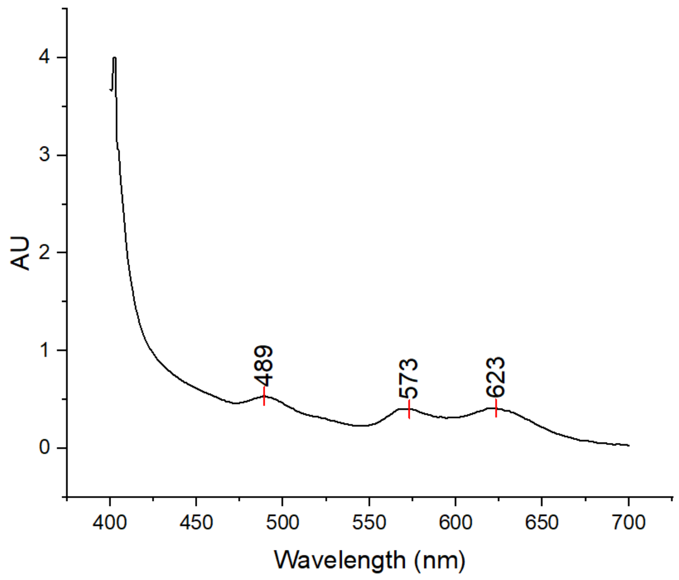

Figure S16. UV-vis absorbance spectrum of **G1⊂M3** (10 μM in DMSO).

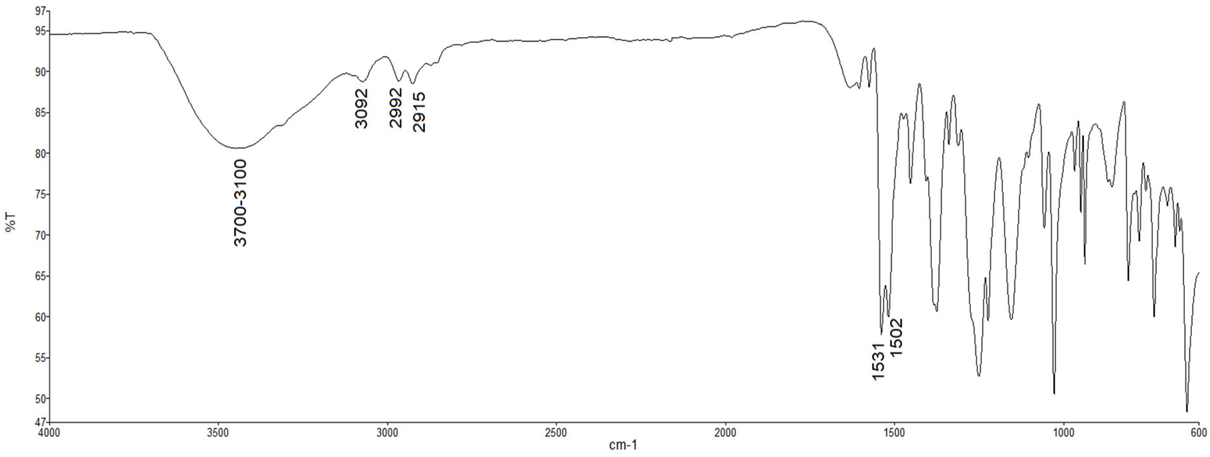

Figure S17. ATR FT-IR spectrum spectrum of **G1⊂M3**.

1.3 Synthesis of G2⊂M1

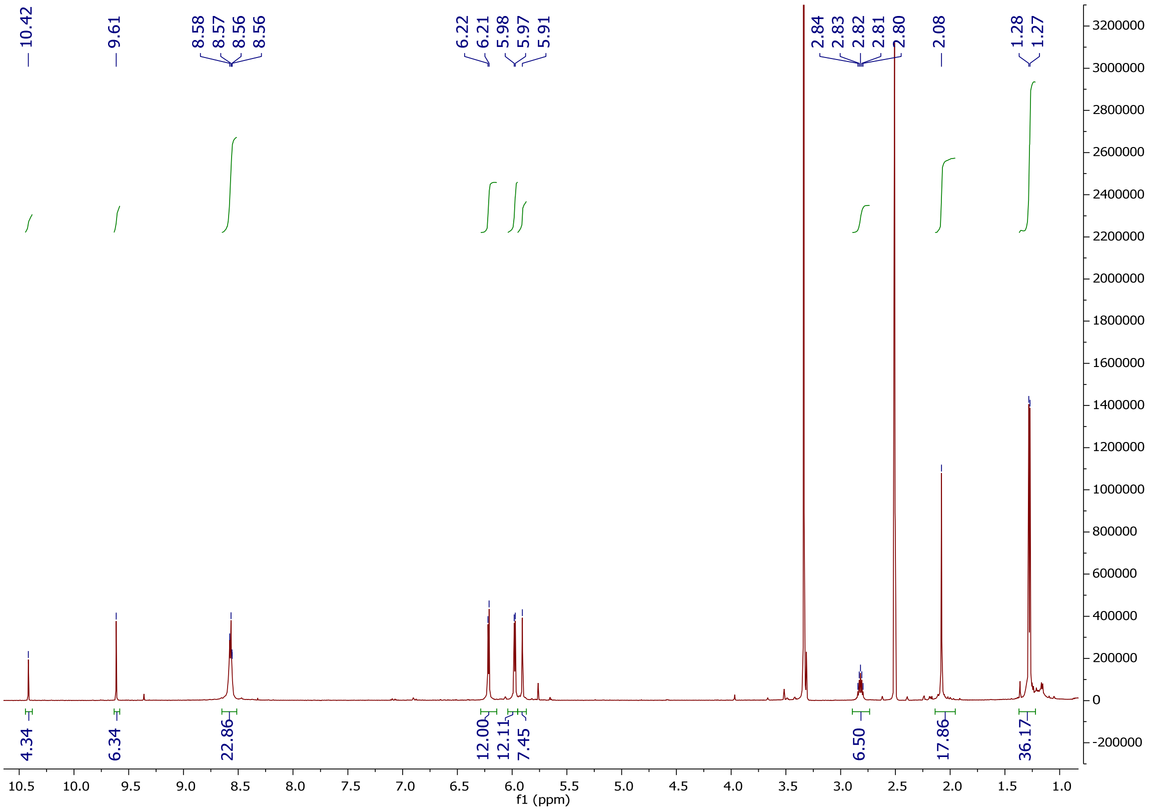

Figure S18. ^{1}H NMR spectrum of **G2⊂M1** in DMSO-d_6 at 25 °C.

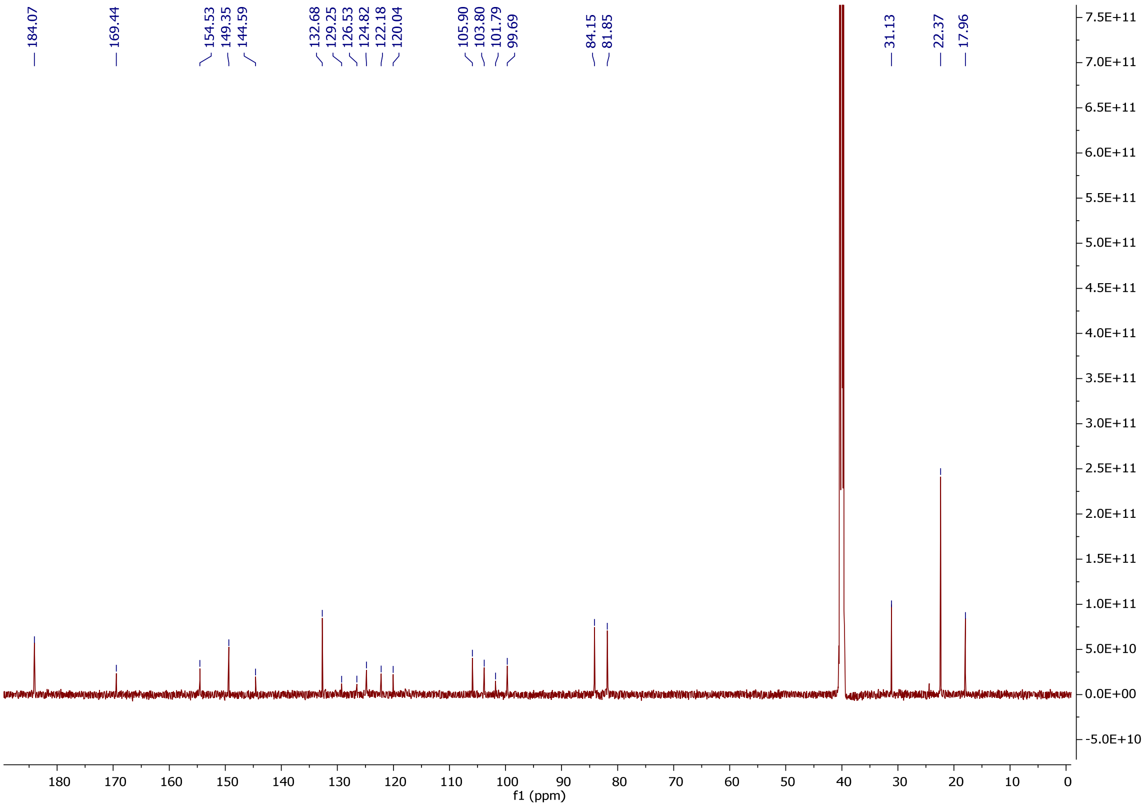

Figure S19. ^{13}C NMR spectrum of **G2⊂M1** in DMSO-d_6 at 25 °C.

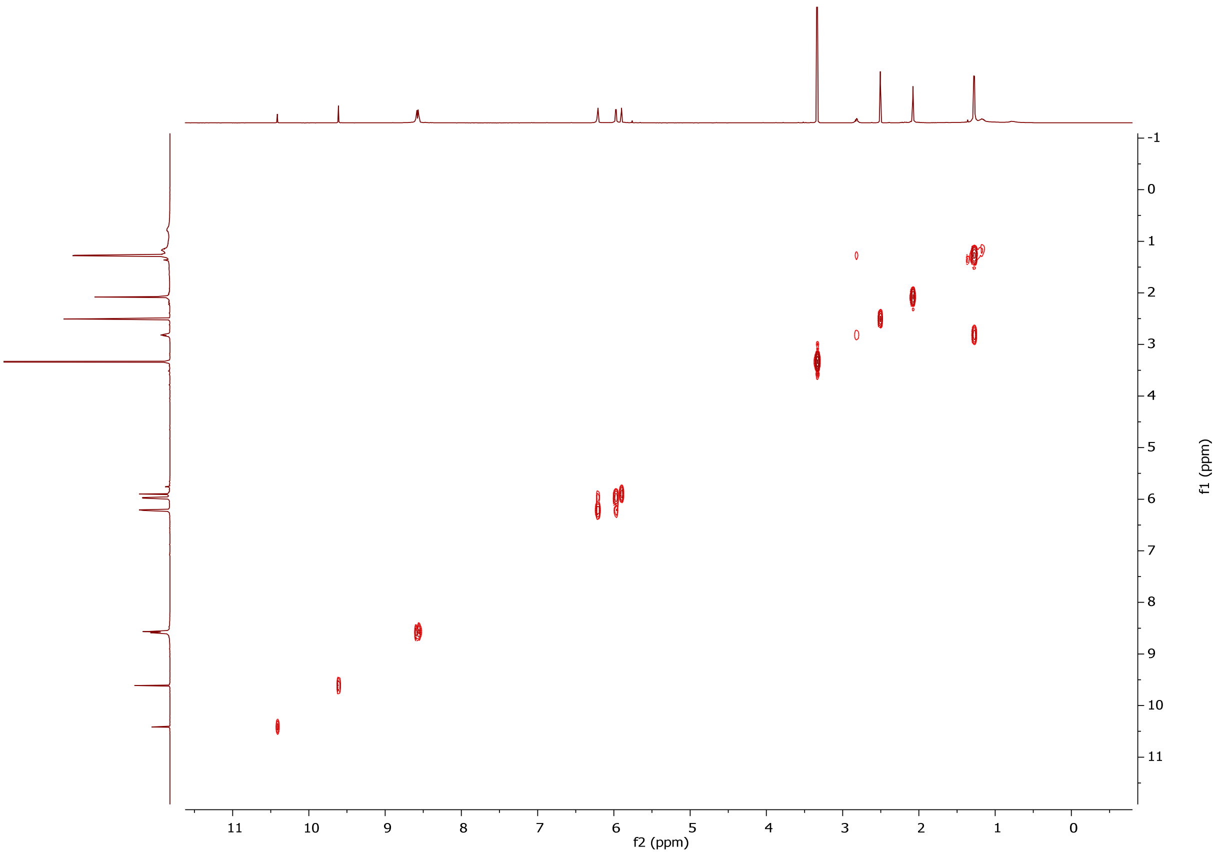

Figure S20. ^1H-^1H COSY NMR spectrum of **G2⊂M1** in DMSO-d_6 at 25 °C.

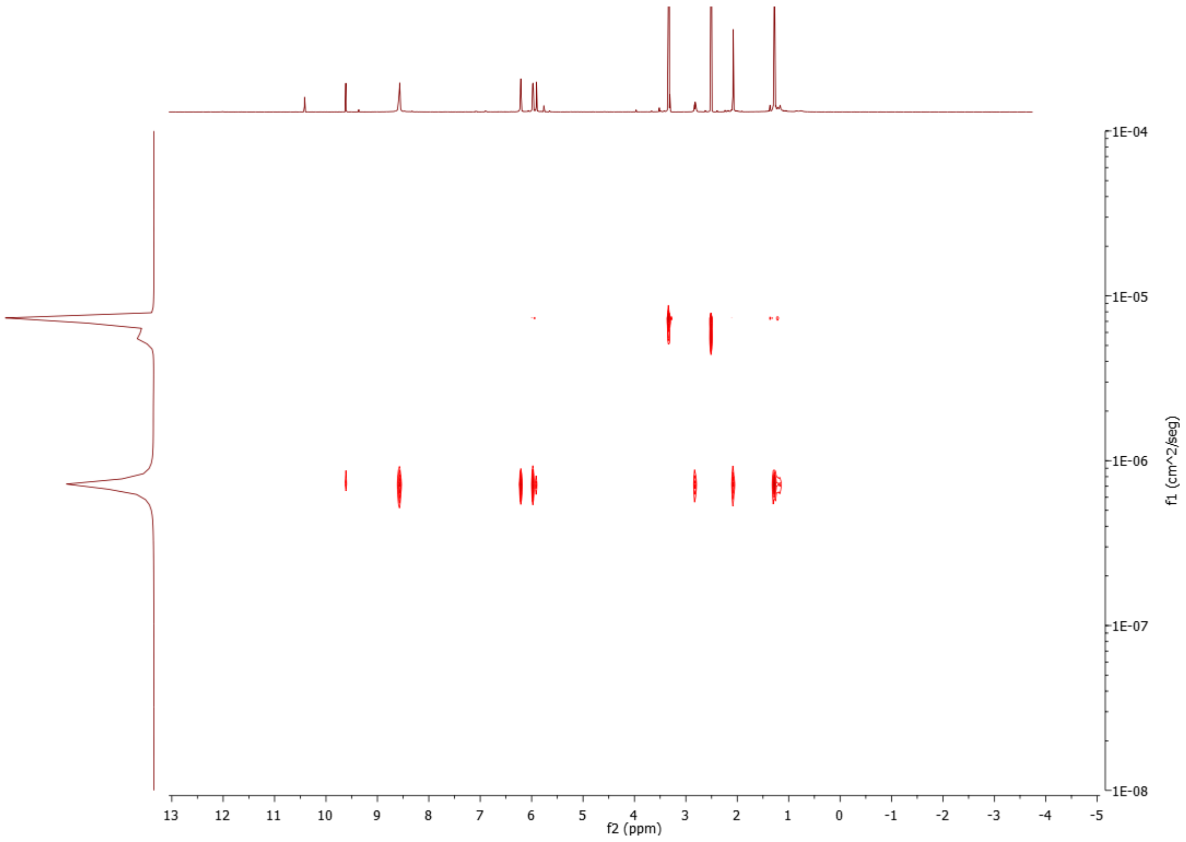

Figure S21. DOSY NMR spectrum of **G2⊂M1** in DMSO-d_6 at 25 °C.

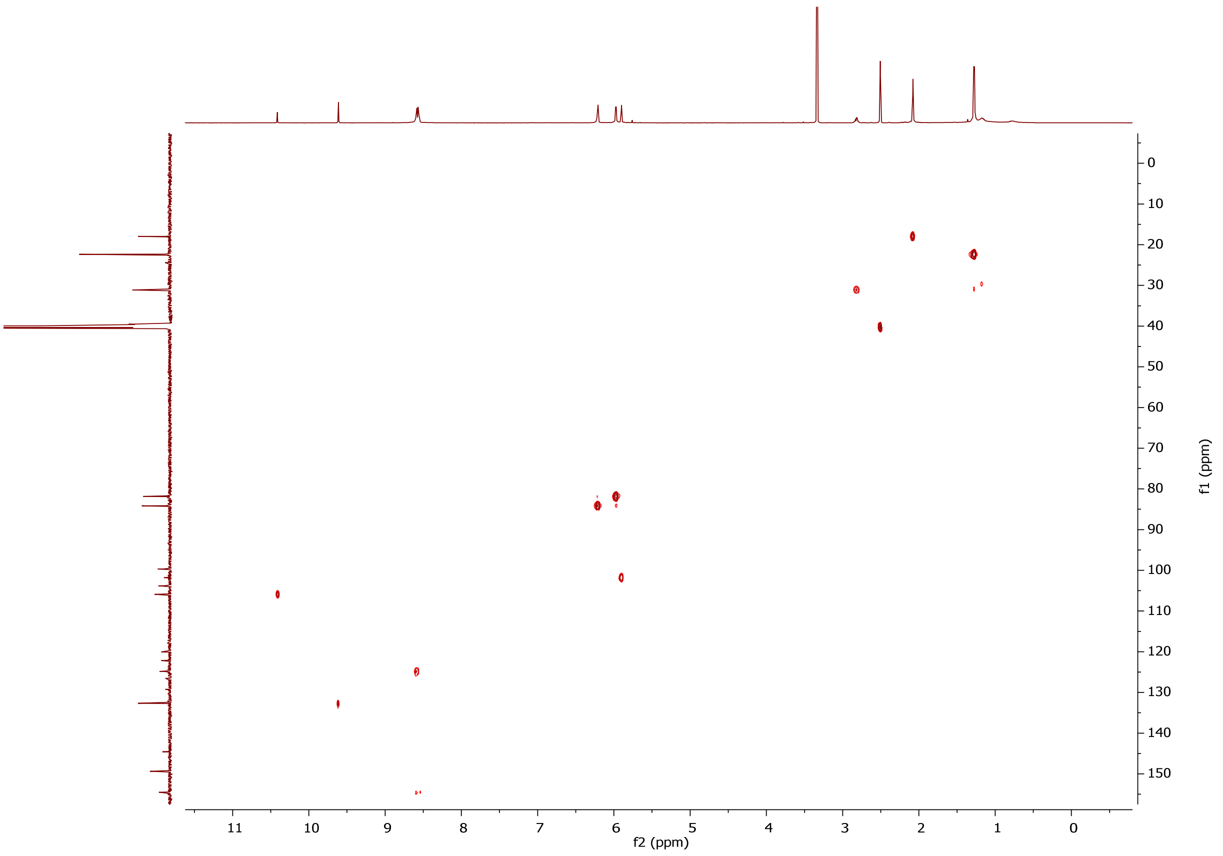

Figure S22. ^{1}H-^{13}C HSQC NMR spectrum of **G2⊂M1** in DMSO-d_6 at 25 °C.

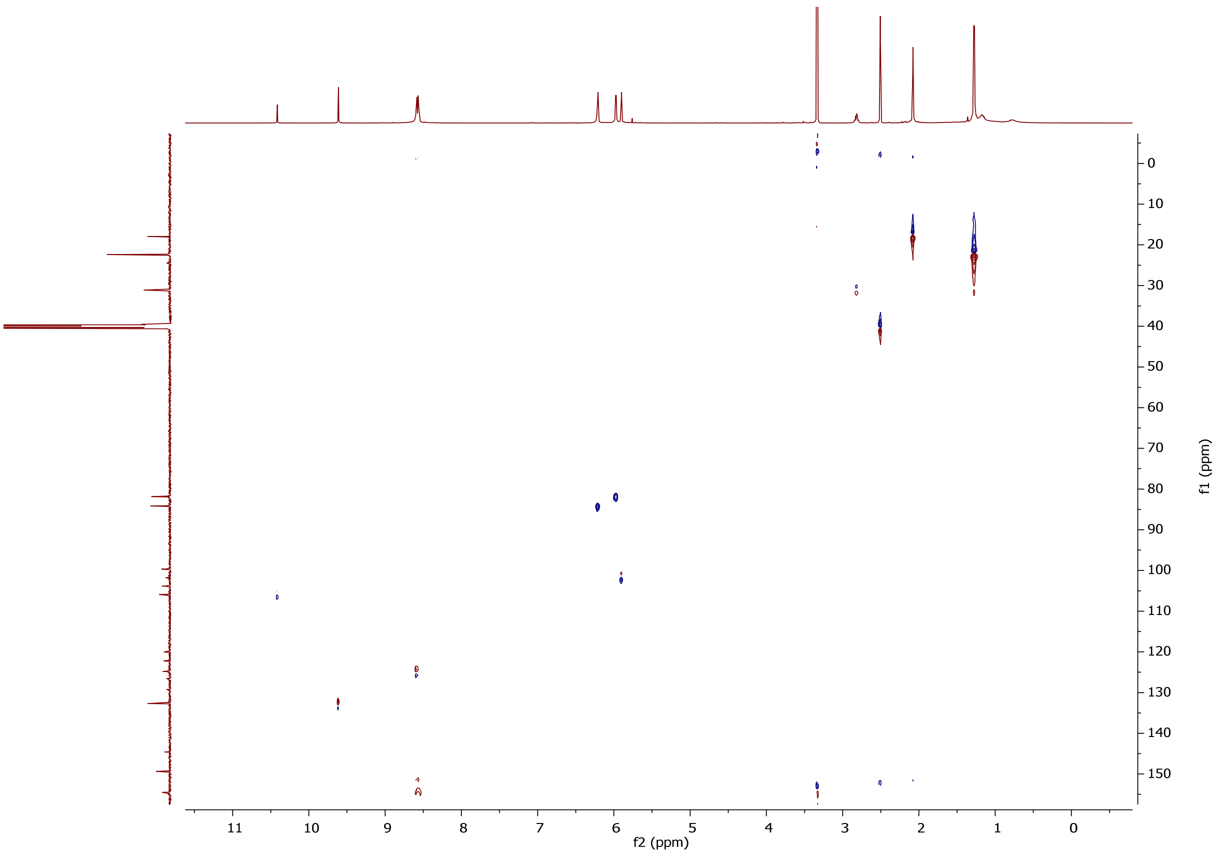

Figure S23. ^{1}H-^{13}C HMQC NMR spectrum of **G2⊂M1** in DMSO-d_6 at 25 °C.

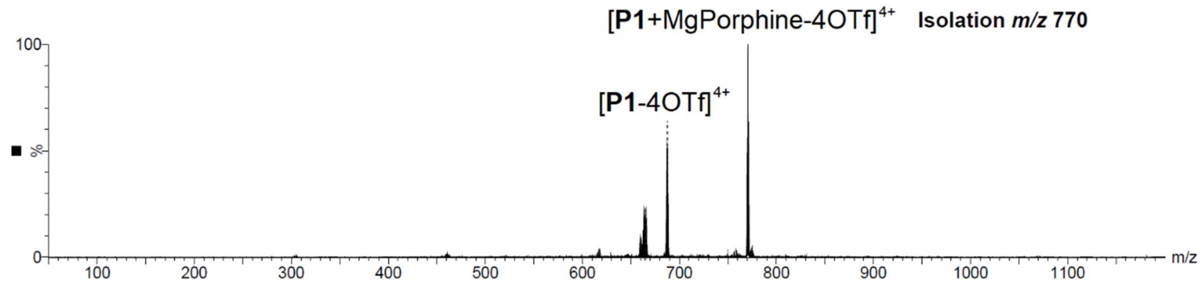

Figure S24. ESI-MS spectrum of **G2⊂M1**.

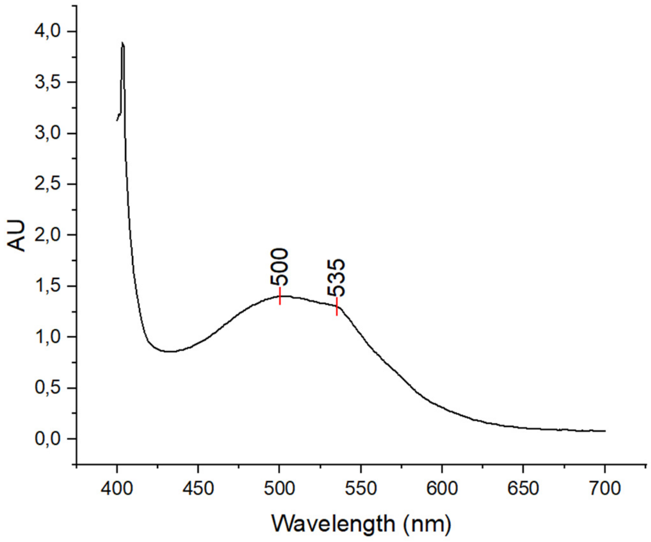

Figure S25. UV-vis absorbance spectrum of **G2⊂M1** (10 μM in DMSO).

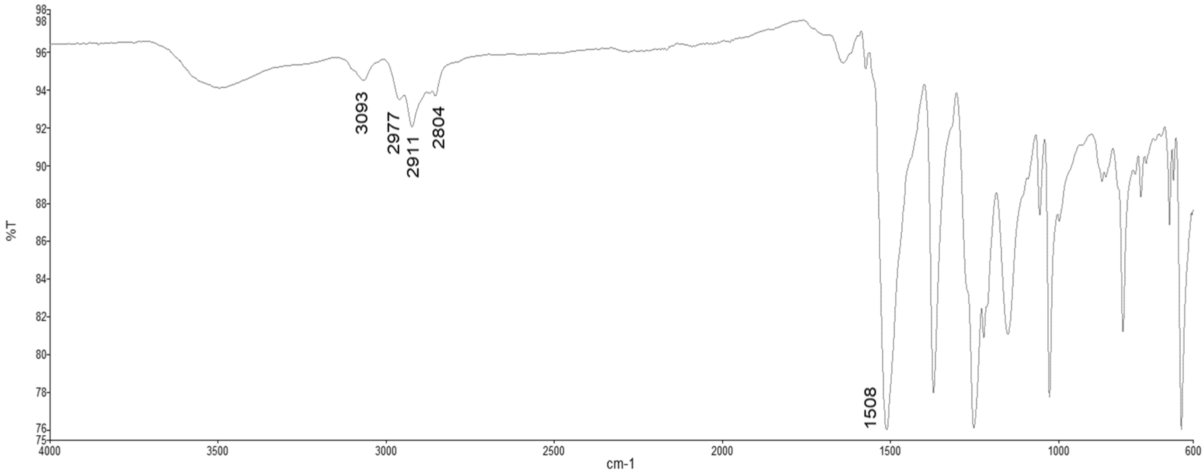

Figure S26. ATR FT-IR spectrum spectrum of **G2⊂M1**.

1.4 Synthesis of G2⊂M4

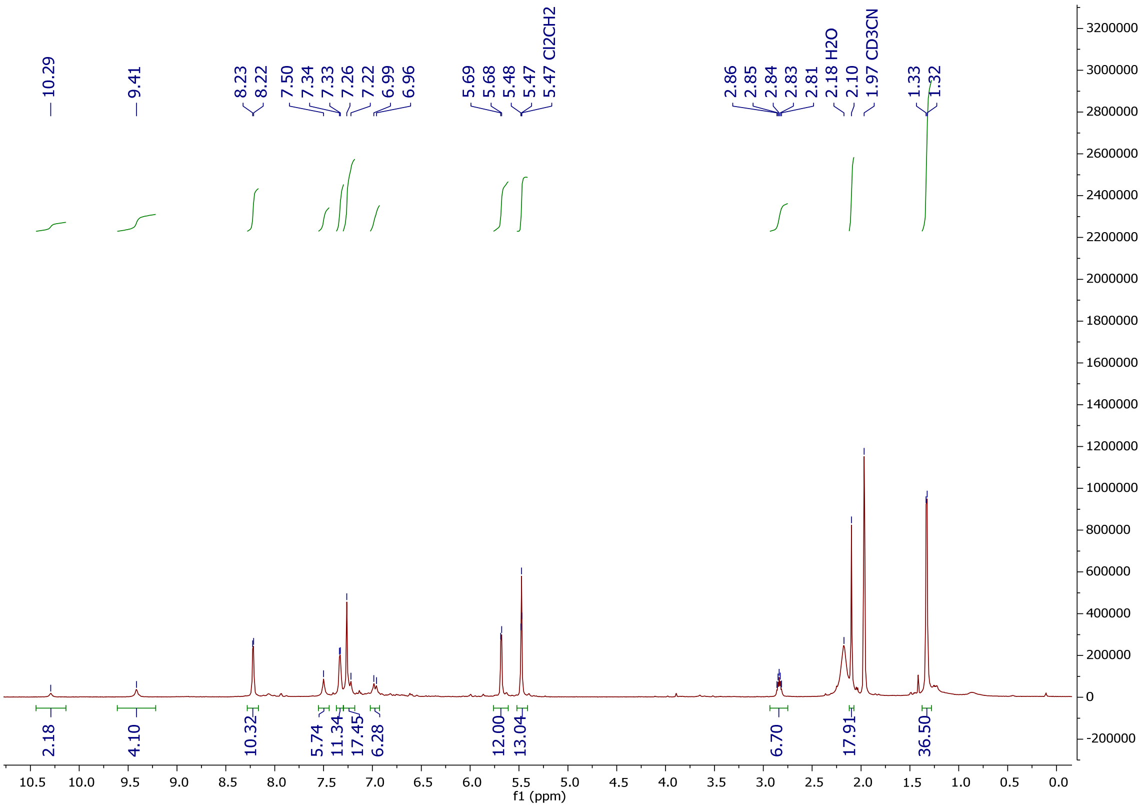

Figure S27. ^{1}H NMR spectrum of **G2⊂M4** in CD_3CN at 25 °C.

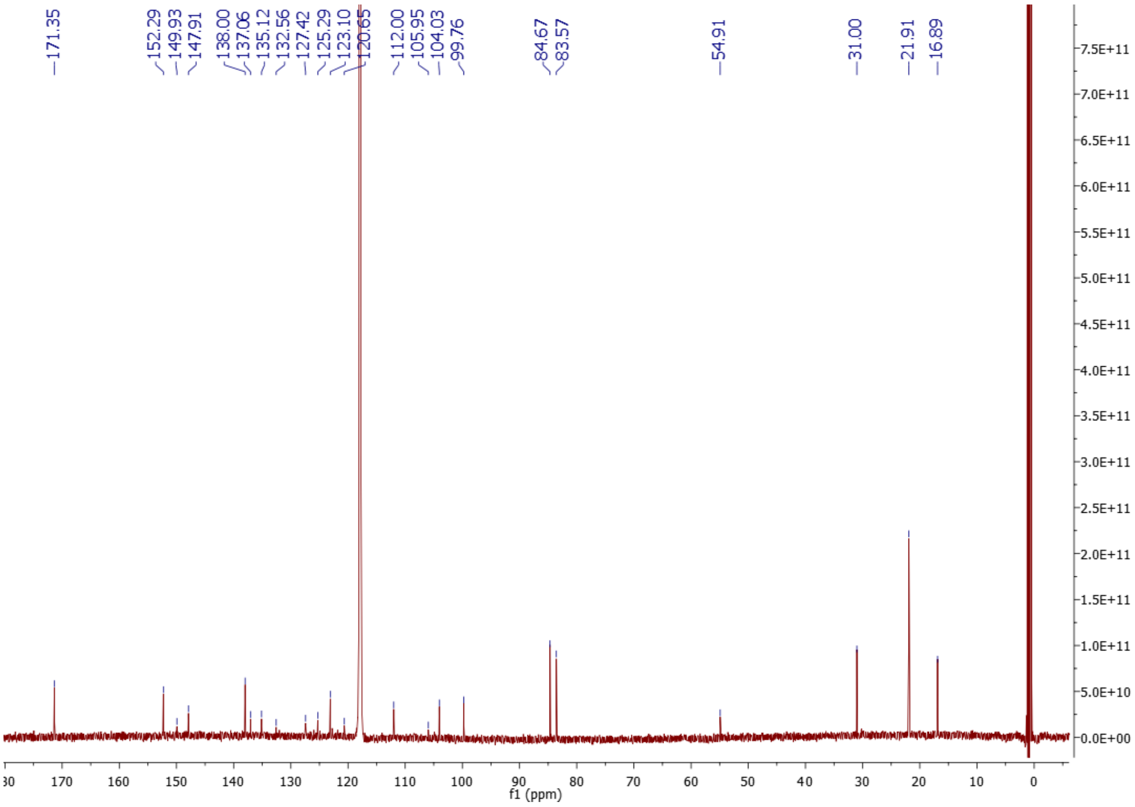

Figure S28. ^{13}C NMR spectrum of **G2⊂M4** in CD_3CN at 25 °C.

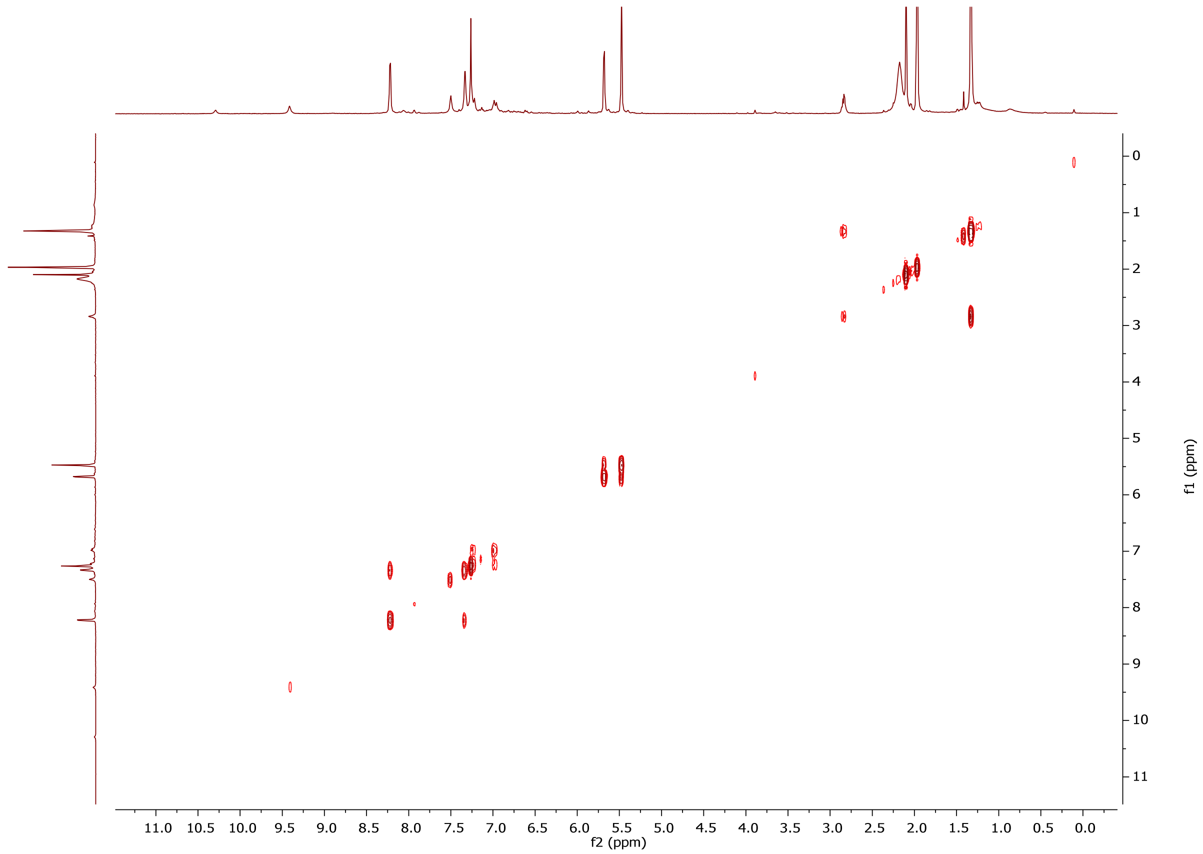

Figure S29. ¹H-¹H COSY NMR spectrum of **G2⊂M4** in CD_3CN at 25 °C.

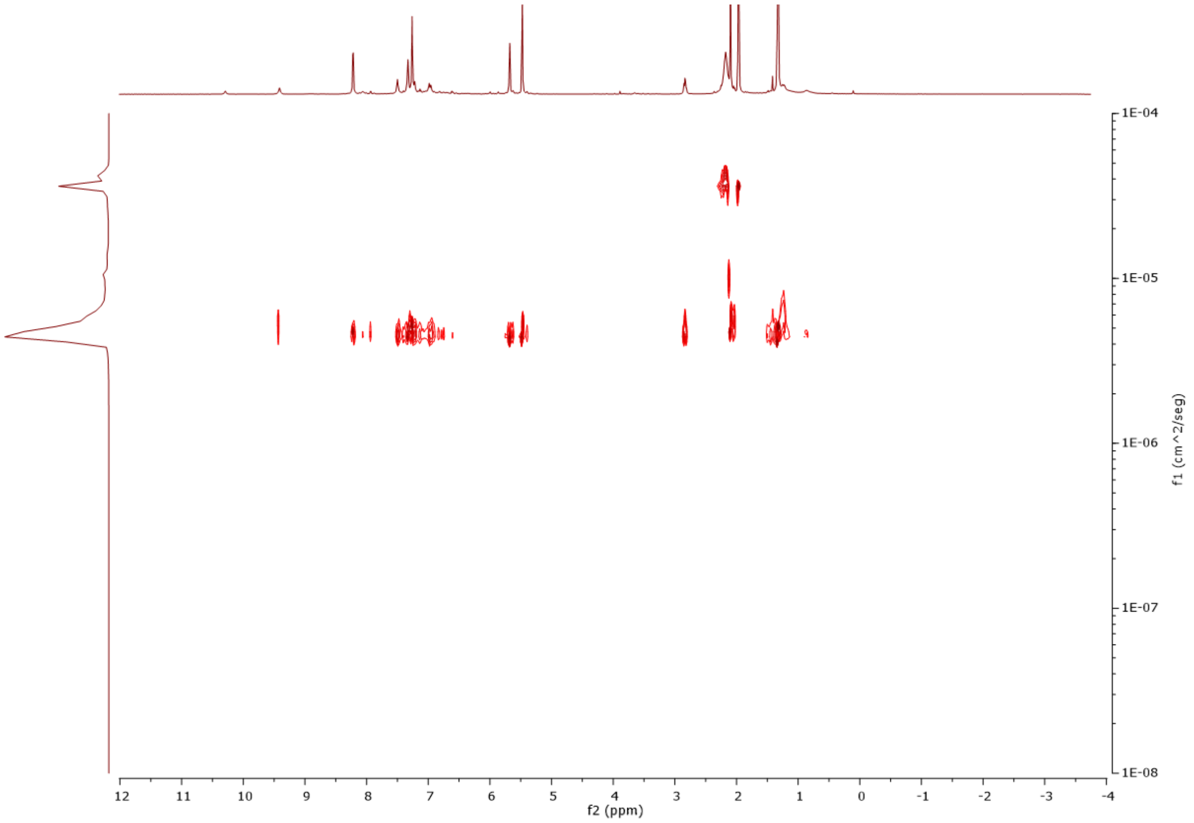

Figure S30. DOSY NMR spectrum of **G2⊂M4** in CD_3CN at 25 °C.

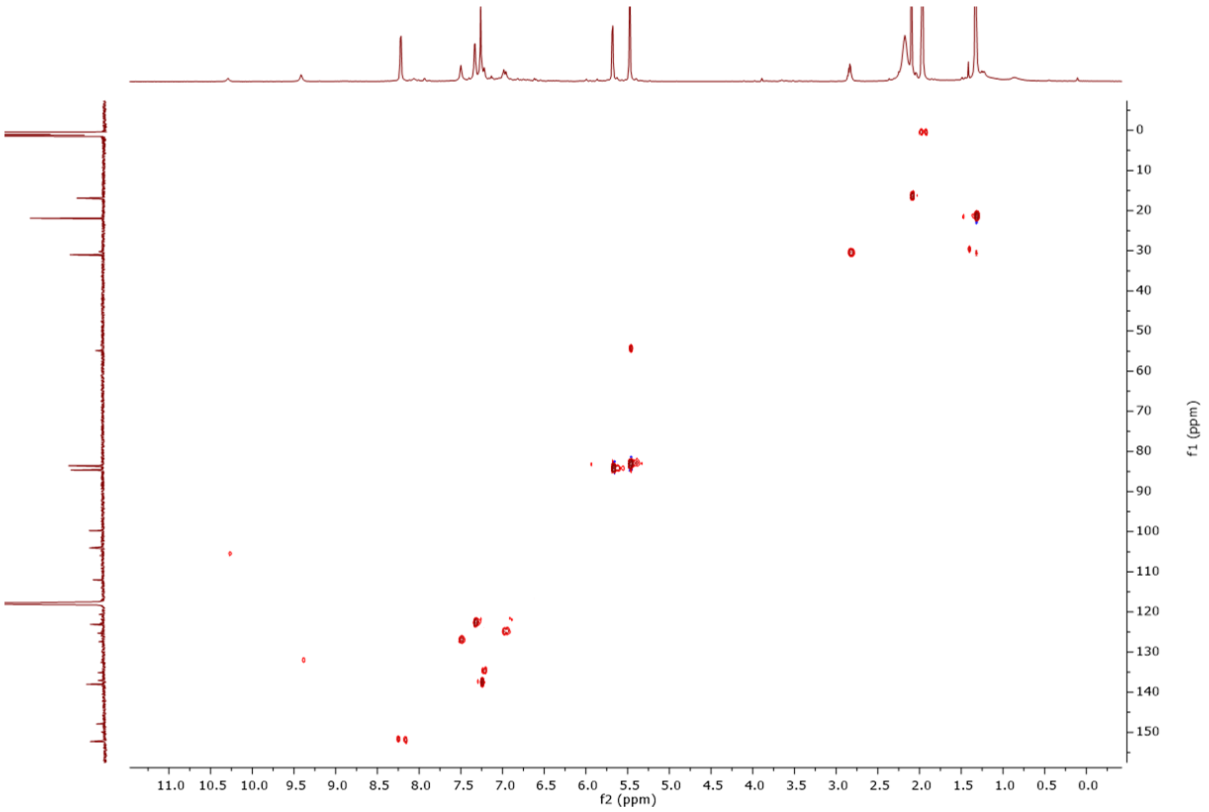

Figure S31. ^{1}H-^{13}C HSQC NMR spectrum of **G2⊂M4** in CD_3CN at 25 °C.

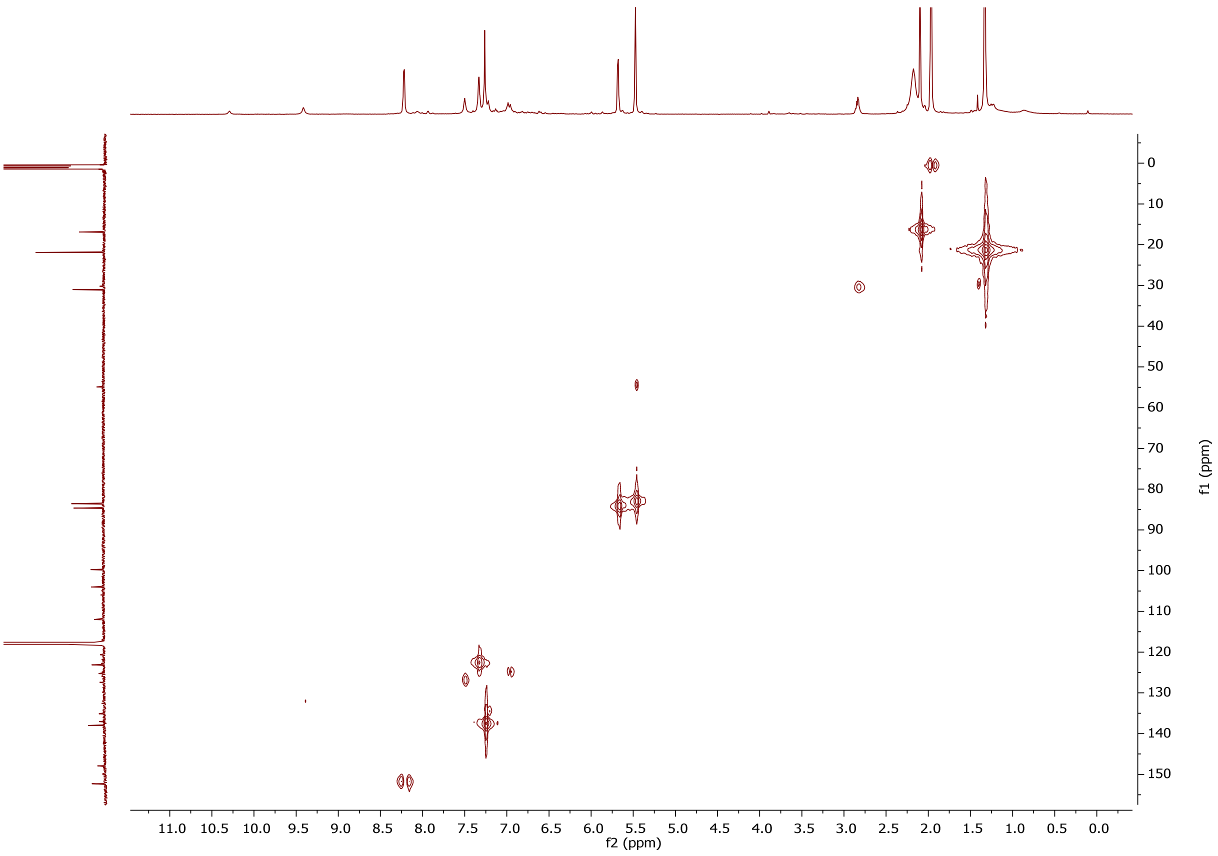

Figure S32. ^{1}H-^{13}C HMQC NMR spectrum of **G2⊂M4** in CD_3CN at 25 °C.

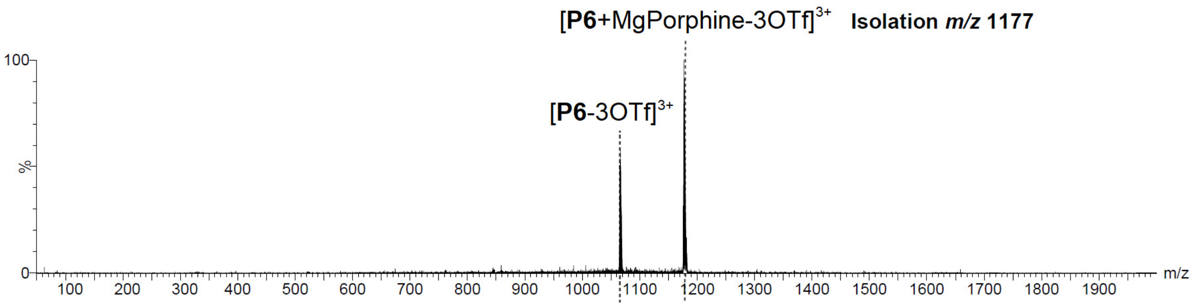

Figure S33. ESI-MS spectrum of **G2⊂M4**.

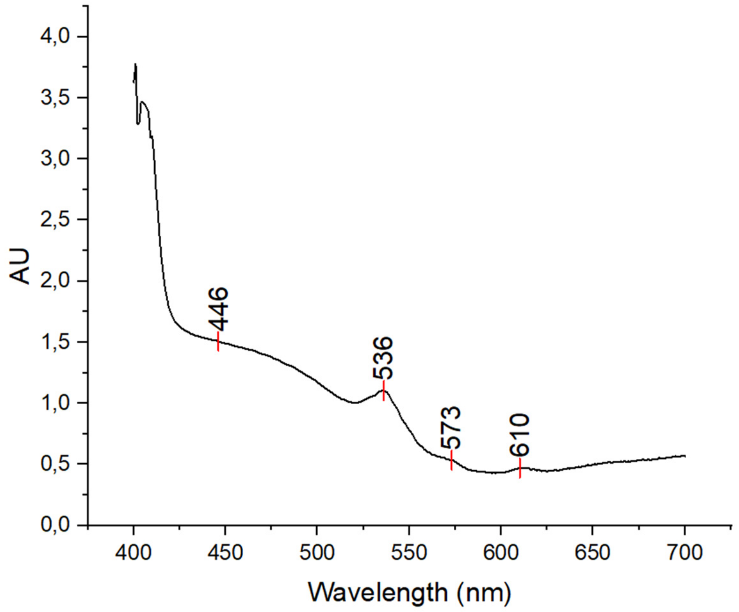

Figure S34. UV-vis absorbance spectrum of **G2⊂M4** (10 µM in DMSO).

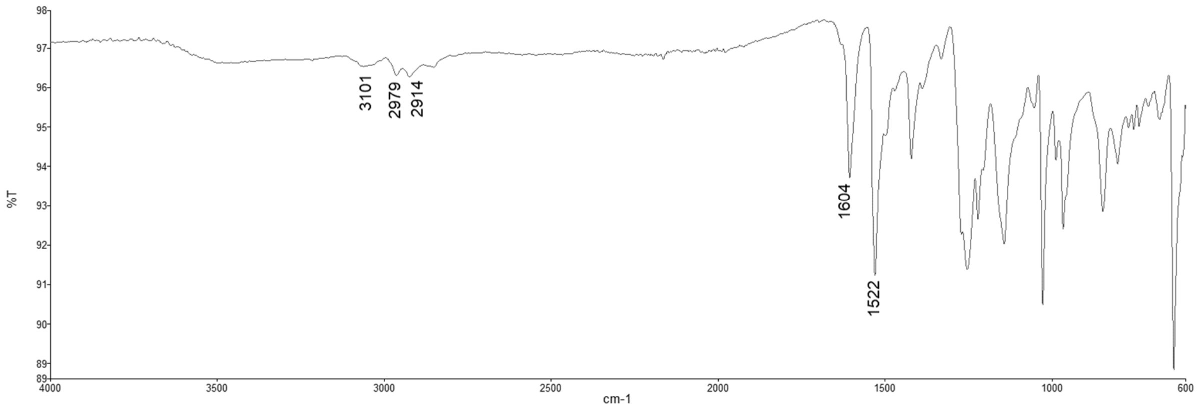

Figure S35. ATR FT-IR spectrum spectrum of **G2⊂M4**.

1.5 Synthesis of G2⊂M6

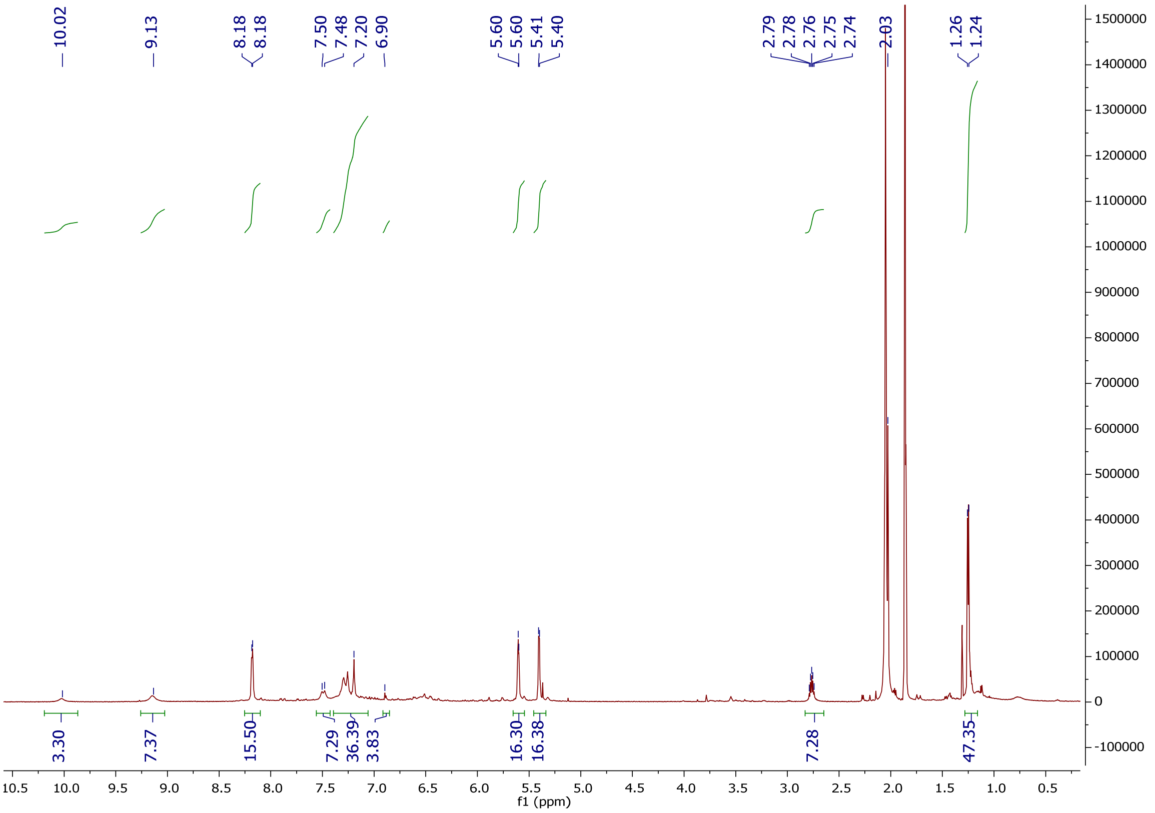

Figure S36. ^{1}H NMR spectrum of **G2⊂M6** in CD_3CN at 25 °C.

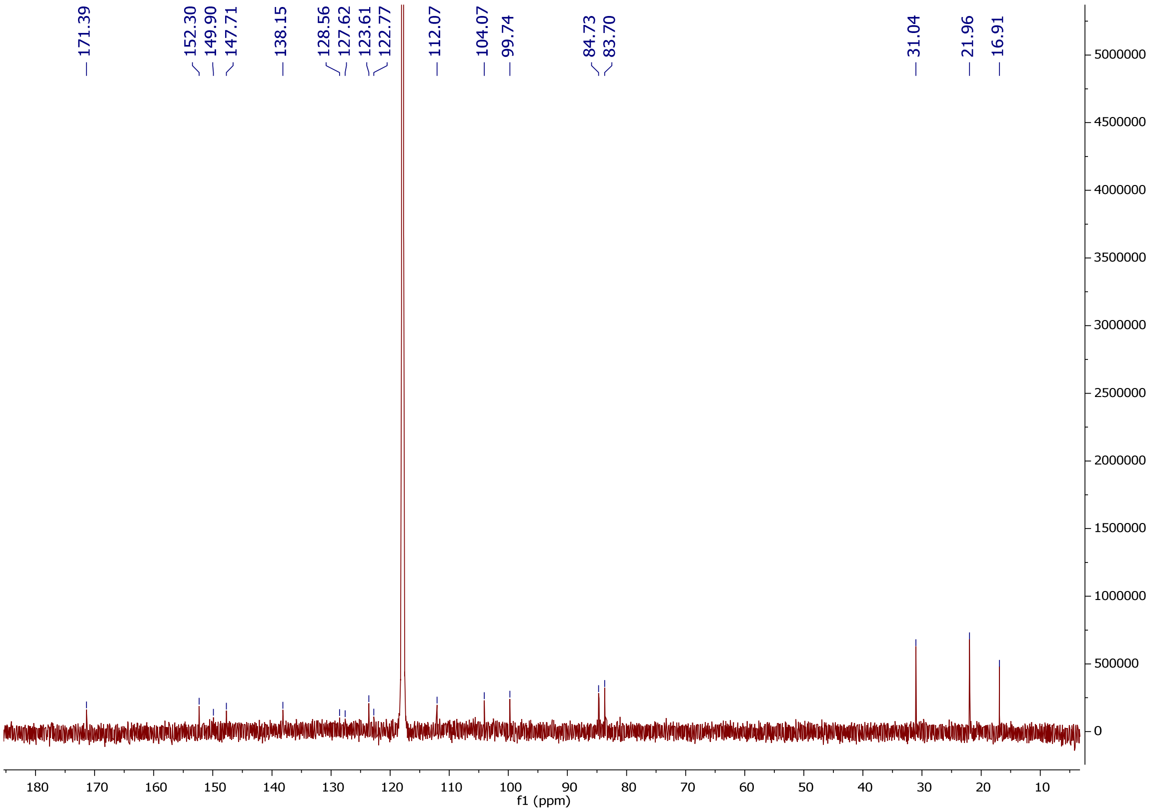

Figure S37. ^{13}C NMR spectrum of **G2⊂M6** in CD_3CN at 25 °C.

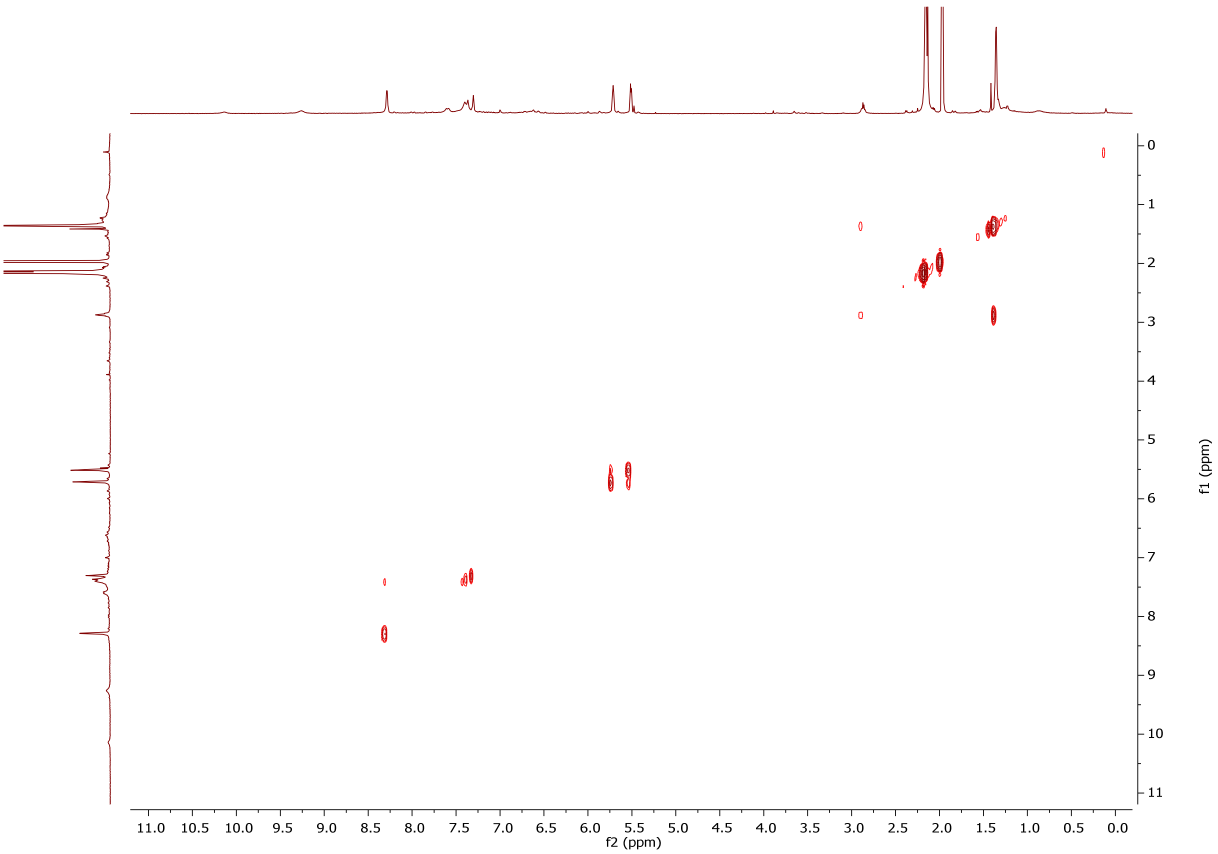

Figure S38. ¹H-¹H COSY NMR spectrum of **G2⊂M6** in CD_3CN at 25 °C.

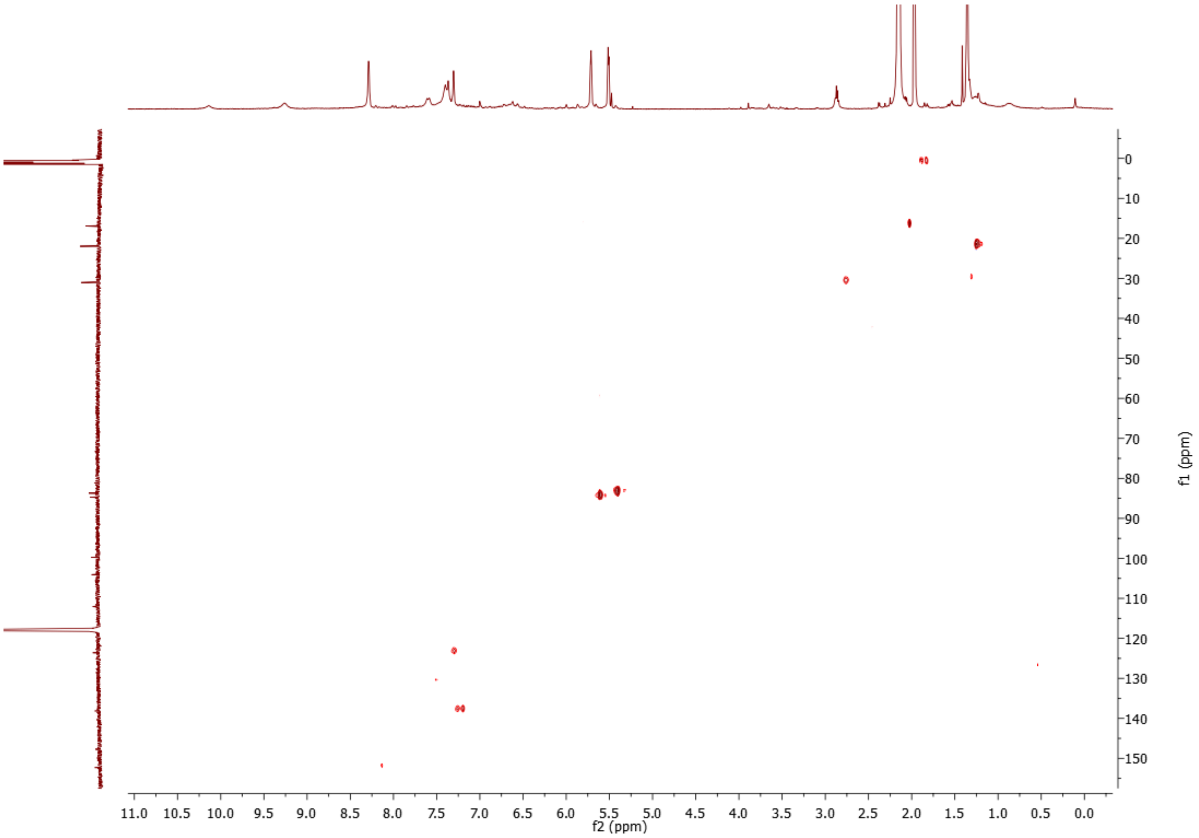

Figure S39. ¹H-¹³C HSQC NMR spectrum of **G2⊂M6** in CD_3CN at 25 °C.

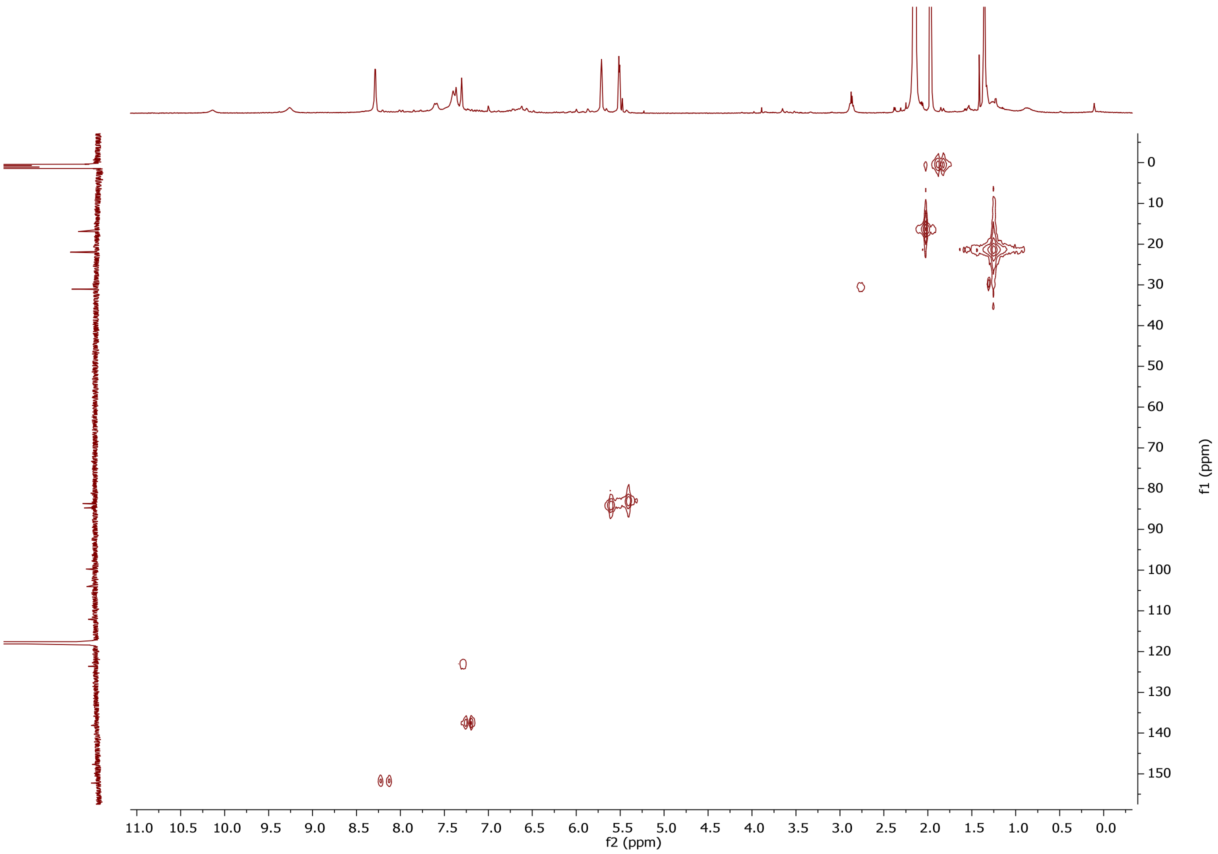

Figure S40. ^{1}H-^{13}C HMQC NMR spectrum of **G2⊂M6** in CD_3CN at 25 °C.

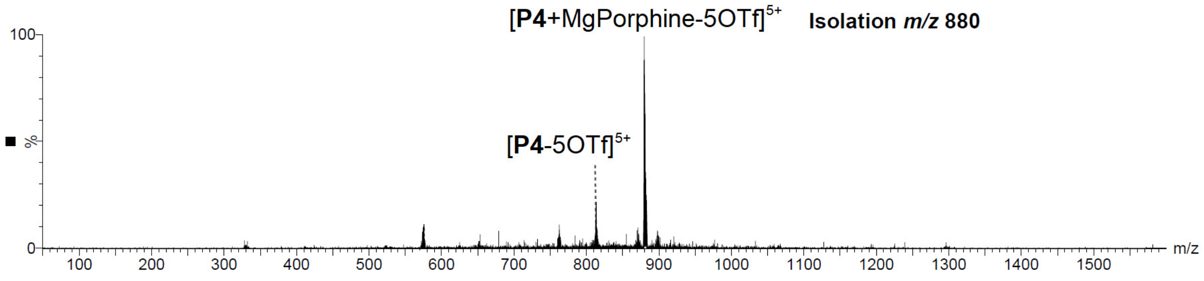

Figure S41. ESI-MS spectrum of **G2⊂M6**.

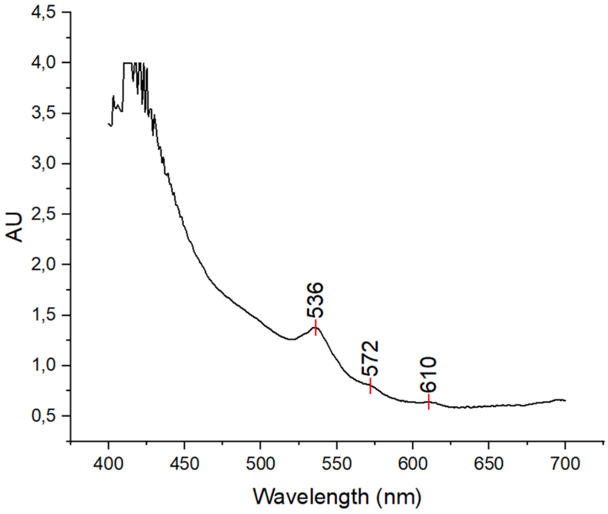

Figure S42. UV-vis absorbance spectrum of **G2⊂M6** (10 μM in DMSO).

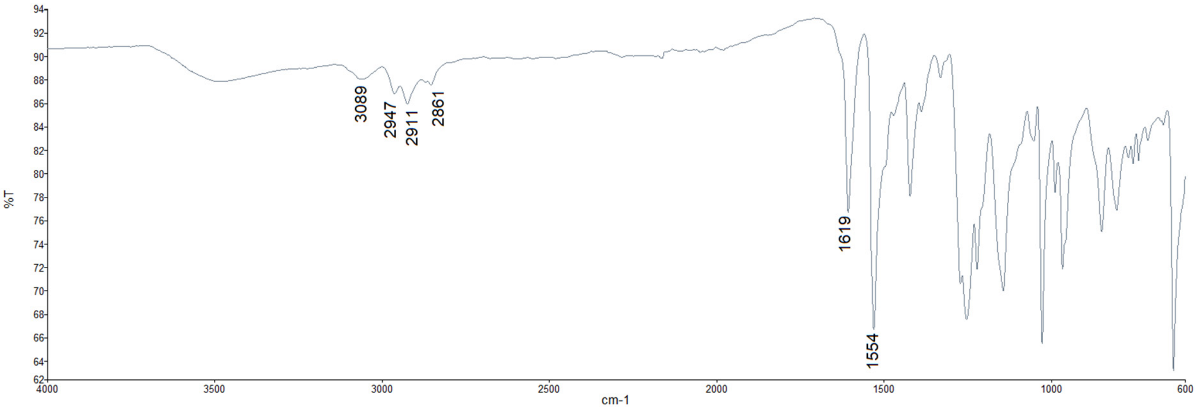

Figure S43. ATR FT-IR spectrum spectrum of **G2⊂M6**.
